# Effects of different velocity loss thresholds during resistance training on jump and sprint performance in trained populations: a systematic review and three-level meta-analysis

**DOI:** 10.3389/fphys.2026.1893020

**Published:** 2026-10-06

**Authors:** Jie Zhang, Shan Zhang, Yan Cui, Yijie Huang, Yiran Dan, Xiuling Wu, Xing Ye, Hao Zeng, Bitai Wu, Mingyue Yin

**Affiliations:** 1 School of Physical Education and Tourism, Polus International College, Chengdu, Sichuan, China; 2 School of Physical Education, Chengdu Sport University, Chengdu, Sichuan, China; 3 School of Physical Education, Sichuan University of Science & Engineering, Zigong, China; 4 School of Leisure Sports, Chengdu Sport University, Chengdu, Sichuan, China; 5 School of Sport and Human Movement Science, Beijing Sport University, Beijing, China; 6 School of Wushu, Tianjin University of Sport, Tianjin, China; 7 School of Physical Education and Sports Science, Hengyang Normal University, Hunan, China; 8 School of Coaching, Shanghai University of Sport, Shanghai, China

**Keywords:** jump performance, sprint performance, three-level meta-analysis, velocity loss threshold, velocity-based resistance training

## Abstract

**Purpose:**

To systematically evaluate the effects of different velocity loss (VL) thresholds during resistance training on jump and sprint performance in trained populations and to examine potential moderators.

**Methods:**

PubMed, Web of Science, Embase, the Cochrane Library, and SPORTDiscus were searched from inception through April 10, 2026. Randomized controlled trials meeting the prespecified PICOS criteria were included. A three-level random-effects meta-analysis was conducted to calculate Hedges’ g and 95% confidence intervals (CIs), accounting for dependence among multiple effect sizes within studies. Risk of bias and certainty of evidence were assessed using RoB 2 and GRADE, respectively.

**Results:**

Twelve randomized controlled trials comprising 42 effect sizes were included. Lower VL thresholds produced a small but statistically significant favorable effect on combined jump and sprint performance compared with higher VL thresholds (g=0.29, 95% CI: 0.17 to 0.42, p<0.001), with negligible heterogeneity ( ${\text{I}}_{\text{total}}^{2}=0.1\%$). Domain-specific analyses showed significant favorable effects for both jump performance (g=0.27, 95% CI: 0.10 to 0.45, p=0.002) and sprint performance (g=0.32, 95% CI: 0.13 to 0.50, p<0.001). Outcome-specific analyses indicated clearer effects for countermovement jump, 10 m sprint, and 30 m sprint, whereas the estimates for squat jump and 20 m sprint were not statistically significant. Significant effects were observed for the 10% versus 30% VL and 10% versus 20% VL comparisons, whereas the 20% versus 40% VL comparison yielded a smaller, non-significant effect. Exploratory moderator analyses did not detect statistically significant effects of population type, training frequency, training mode, intervention duration, or VL-threshold difference; however, these analyses may have been underpowered because of the limited number of included studies. The direction and statistical significance of the pooled effect were robust across alternative pre–post correlation assumptions and model specifications, although heterogeneity estimates and prediction intervals were more sensitive to higher assumed correlations. No evidence of significant small-study effects was detected.

**Conclusions:**

Lower VL thresholds may confer small favorable effects on jump and sprint performance in trained populations, with clearer evidence for countermovement jump and selected sprint outcomes. However, the effects were not consistent across all outcomes, and the certainty of evidence ranged from low to moderate. Lower VL may therefore be considered as one potential option for speed–power-oriented resistance training rather than a universally superior prescription.

**Systematic review registration:**

https://www.crd.york.ac.uk/PROSPERO/, identifier CRD420261396083.

## 1 Introduction

Jump and sprint performance are key determinants of athletic performance in sports requiring rapid acceleration, deceleration, jumping, changes of direction, and high power output over short durations. Lower-limb neuromuscular function is therefore closely associated with sport-specific performance and competitive success (Suchomel et al., 2018, 2016). Resistance training is widely used to enhance maximal strength, power, and lower-limb mechanical capacities (Makaruk et al., 2024; McGuigan et al., 2012), and previous evidence indicates that it can improve strength, jump performance, and linear sprint ability (Lesinski et al., 2016; Ulloa-Sánchez et al., 2025). Optimizing the prescription and monitoring of resistance training is therefore important for the development of speed–power performance.

Velocity-based resistance training (VBT) has emerged as an important complement to traditional percentage-based resistance training. Rather than relying exclusively on predetermined loads and fixed repetition schemes, VBT uses movement velocity to monitor training intensity, fatigue status, and daily performance fluctuations, thereby supporting real-time adjustment of the training load (González-Badillo and Sánchez-Medina, 2010; Weakley et al., 2021). Previous systematic reviews suggest that VBT can improve maximal strength, jump performance, and sprint performance in trained individuals, while potentially supporting more flexible and individualized training prescription (Baena-Marín et al., 2022; Held et al., 2022; Orange et al., 2022; Włodarczyk et al., 2021).

Within the VBT framework, the velocity loss threshold (VL) is commonly used to regulate within-set training volume and fatigue exposure. VL generally refers to the permitted reduction in movement velocity relative to the fastest repetition within a set and can serve as a criterion for terminating the set (Jukic et al., 2023; Sánchez-Medina and González-Badillo, 2011). Lower VL thresholds typically result in earlier set termination and fewer repetitions, whereas higher thresholds allow greater repetition volume and fatigue accumulation (González-Badillo et al., 2017; Sánchez-Medina and González-Badillo, 2011). Accordingly, different VL thresholds may produce distinct training stimuli and potentially influence subsequent strength and speed–power adaptations (Hernández-Belmonte and Pallarés, 2022; Jukic et al., 2023).

Several systematic reviews and meta-analyses have examined adaptations associated with different VL thresholds, but their findings are not entirely consistent. Existing evidence generally suggests that low-to-moderate VL thresholds may be more favorable for jump, sprint, and other velocity-oriented outcomes, whereas the additional volume and fatigue associated with higher thresholds do not necessarily produce superior speed–power adaptations (Chen et al., 2024; Hernández-Belmonte and Pallarés, 2022; Jukic et al., 2023). Some evidence suggests that VL thresholds of ≤20% may be suitable for countermovement jump performance, whereas thresholds of ≤10% may be more appropriate for sprint performance (Chen et al., 2024). However, network meta-analytic evidence indicates that both low and high VL approaches may be beneficial compared with other resistance training strategies, while their relative superiority remains uncertain (Held et al., 2022). Dose–response findings have also suggested that 20%–30% VL may be favorable for maximal strength development, although this evidence has focused primarily on one-repetition maximum outcomes rather than jumping and sprinting (Zhang et al., 2023).

Existing reviews of VBT and VL have several limitations. First, many have focused on comparisons between VBT and traditional percentage-based training rather than directly examining differences between VL thresholds (Held et al., 2022; Liao et al., 2021; Orange et al., 2022). Second, strength, jump, sprint, and other performance outcomes have often been pooled together, limiting conclusions regarding specific outcomes such as countermovement jump, squat jump, and sprint performance over different distances. Third, individual studies frequently report multiple outcomes, VL groups, or subgroup comparisons, resulting in statistically dependent effect sizes. Conventional two-level meta-analytic models may not adequately account for this dependence, whereas three-level models can simultaneously estimate sampling error, within-study variance, and between-study variance (Assink and Wibbelink, 2016).

Therefore, this systematic review and three-level meta-analysis aimed to evaluate the effects of lower versus higher VL thresholds during resistance training on jump and sprint performance in trained populations. We also explored whether outcome domain, specific performance outcome, VL comparison type, participant characteristics, and training-related variables moderated the observed effects. The novelty of this study lies in directly comparing lower versus higher VL thresholds in trained populations while applying a three-level meta-analytic framework to account for multiple dependent effect sizes reported within studies. This approach provides outcome-specific evidence regarding the potential role and practical boundaries of VL-based resistance training prescription for jump and sprint performance.

## 2 Materials and methods

### 2.1 Protocol and registration

This systematic review and meta-analysis was conducted in accordance with the Preferred Reporting Items for Systematic Reviews and Meta-Analyses (PRISMA) statement and the Cochrane Handbook for Systematic Reviews of Interventions. The study protocol was prospectively registered in the International Prospective Register of Systematic Reviews (PROSPERO; registration number: CRD420261396083).

### 2.2 Search strategy

Before the formal literature search, a preliminary search was conducted to refine the search terms and optimize the search strategy. Two researchers independently searched PubMed, Web of Science, Embase, Cochrane Library, and SPORTDiscus from inception to April 10, 2026. The search strategy combined controlled vocabulary terms, where applicable, and free-text terms related to “velocity loss,” “velocity-based training,” “resistance training,” “jump,” “sprint,” and “athletic performance,” using Boolean operators. In addition to electronic database searches, the reference lists of included studies and relevant systematic reviews were manually screened to minimize the risk of missing eligible studies. The detailed search strategy is provided in **Supplementary Table S2**.

### 2.3 Study selection and eligibility criteria

Two researchers independently screened titles, abstracts, and full texts according to predefined inclusion and exclusion criteria. Disagreements during the selection process were resolved through discussion, and a third researcher was consulted when necessary. EndNote 21 was used for reference management and duplicate removal. The eligibility criteria were developed according to the PICOS framework, including participants, intervention, comparison, outcomes, and study design (**Supplementary Table S3**).

Trained populations were defined as competitive athletes with a documented sport-specific training background or non-athletes with at least one year of regular resistance-training experience. Youth samples were eligible only when the original study clearly documented organized sport-specific and/or resistance-training experience. Untrained individuals and clinical or rehabilitation populations were excluded. Because this review focused on the relative effects of lower versus higher VL thresholds within previously trained samples, eligible athlete and trained non-athlete populations were pooled, while population type was additionally examined as a potential moderator. Detailed training backgrounds for each included sample are presented in **Supplementary Table S4**.

### 2.4 Risk of bias and certainty of evidence assessment

The risk of bias of the included randomized controlled trials was assessed using the latest Cochrane Risk of Bias 2 tool (RoB 2) (Sterne et al., 2019). RoB 2 evaluates five domains: the randomization process (D1), deviations from intended interventions (D2), missing outcome data (D3), measurement of the outcome (D4), and selection of the reported result (D5). The overall risk of bias was judged as “low risk,” “some concerns,” or “high risk” (Higgins et al., 2019; Sterne et al., 2019). The certainty of evidence for the main outcomes was assessed using the GRADE approach and summarized with GRADEpro GDT software (GDT; Guyatt et al., 2008). Because all included studies were randomized controlled trials, the initial certainty of evidence was rated as high and was downgraded where appropriate based on risk of bias, inconsistency, indirectness, imprecision, and publication bias (Guyatt et al., 2008). Detailed GRADE judgments and explicit reasons for downgrading the certainty of evidence for each main outcome are provided in **Supplementary Table S7**. All assessments were performed independently by two researchers, and disagreements were resolved through discussion or consultation with a third researcher when necessary.

### 2.5 Data extraction and management

Data from the included studies were extracted and organized using Excel^®^ (Microsoft Corporation, Redmond, WA, USA). The following information was extracted: (1) study characteristics, including first author and publication year; (2) participant characteristics, including sample size, age, sex, training background, and sport type; (3) intervention characteristics, including exercise type, training frequency, intervention duration, training intensity, and VL grouping strategy; (4) VL-related information, including the original VL thresholds and comparison types; and (5) jump and sprint performance outcomes, including countermovement jump (CMJ), squat jump (SJ), reactive strength index (RSI), and 10 m, 15 m, 20 m, and 30 m sprint performance.

Pre- and post-intervention means, standard deviations, and sample sizes were extracted as the basis for effect size calculation. When a study reported multiple outcomes, multiple VL groups, or subgroup-specific results, each eligible effect size was extracted and coded separately. For studies reporting only the standard error of the mean (SEM), standard deviations were calculated using the formula $\text{SD}=\text{SEM}\times \sqrt{N}$. Numerical data were extracted from a graphical presentation for one included study. Specifically, sex-specific countermovement jump data for the 20% and 40% velocity loss groups reported by Rissanen et al. (2022) in Figure 1A were digitized using WebPlotDigitizer. The coordinate axes were calibrated according to the scale presented in the original figure, after which the sex-specific relative changes in CMJ performance and their corresponding standard deviations were extracted. During revision, the graphically extracted values were independently verified by a second researcher against the published figure and were found to be consistent with the values used in the original dataset; therefore, no changes to the corresponding effect-size estimates were required. All other eligible jump and sprint data were obtained directly from numerical values reported in the text or tables of the original studies. Details of the graphical extraction are provided in **Supplementary Table S5**. Other convertible data were processed according to recommended methods. Outcome names, units, and effect directions were standardized during data management. For jump outcomes, higher values were defined as better performance, whereas for sprint time outcomes, lower values were defined as better performance. Data extraction and management were performed independently by two researchers, and disagreements were resolved through discussion or consultation with a third researcher when necessary.

### 2.6 Statistical analysis

For each comparison between different VL thresholds, pre- and post-intervention means, standard deviations, and sample sizes were extracted, and between-group differences in change scores were used as the basis for effect size calculation. When the correlation coefficient between pre-and post-intervention measurements was not reported, a correlation coefficient of r=0.50 was assumed to estimate the standard deviation of the change score (Borenstein et al., 2009; Morris, 2008). To assess the influence of this assumption, effect sizes and corresponding sampling variances for which an imputed pre–post correlation was required were recalculated using alternative correlation coefficients of r=0.30, r=0.70, and r=0.90. The same three-level random-effects model was then refitted under each correlation scenario, and the pooled effect estimates, confidence intervals, prediction intervals, heterogeneity estimates, and statistical significance were compared. All outcomes were treated as continuous variables. Because the included studies used different units and tests for performance outcomes, standardized mean differences based on change scores were calculated as Hedges’ g with small-sample correction (Borenstein et al., 2009; Hedges and Olkin, 1985).

Change scores and their standard deviations were calculated using the following formulas:


$$M_{change}=M_{post}-M_{pre}$$

$$SD_{change}=\sqrt{SD_{pre}^{2}+SD_{post}^{2}-\left(2\times r\times SD_{pre}\times SD_{post}\right)}$$

After calculating change scores for each comparison group, between-group differences in change scores were converted into standardized mean differences (Hedges’ g) and corresponding variances for subsequent meta-analyses. For sprint time outcomes, effect directions were reversed so that improvements in sprint performance corresponded to positive effect sizes. Therefore, all effect sizes were coded such that larger values indicated a more favorable effect.


$$SD_{\text{pooled}}=\sqrt{\frac{\left(n_{1}-1\right)SD_{1}^{2}+\left(n_{2}-1\right)SD_{2}^{2}}{n_{1}+n_{2}-2}}$$

$$ES=\frac{M_{1,\text{change}}-M_{2,\text{change}}}{SD_{\text{pooled}}}\times \left(1-\frac{3}{4\left(n_{1}+n_{2}-2\right)-1}\right)$$

$$SE_{ES}=\sqrt{\frac{n_{1}+n_{2}}{n_{1}\times n_{2}}+\frac{ES^{2}}{2\left(n_{1}+n_{2}\right)}}$$

The corresponding sampling variance was calculated as $v_{ES}=\;SE_{ES}^{2}$.

In the formulas, M represents the mean, SD represents the standard deviation, *SD_change_* represents the standard deviation of the change score, ES represents the effect size, *SE_ES_* represents the standard error of the effect size, *v_ES_* represents the sampling variance of the effect size, n represents the sample size, and *n*_1_ and *n*_2_ represent the sample sizes of the two comparison groups.

Because individual studies frequently reported multiple jump or sprint outcomes, and some studies included multiple VL groups or subgroup comparisons, multiple effect sizes within the same study were likely to be statistically dependent. Therefore, a three-level random-effects meta-analysis was conducted using the rma.mv function in the metafor package, with the random-effects structure specified as random = ~ 1 | study_id/effect_id (Cheung, 2014; Van den Noortgate et al., 2013; Viechtbauer, 2010). Level 1 represented the sampling variance of each effect size, level 2 represented variation among effect sizes within the same study, and level 3 represented variation between studies. Multiple outcomes and VL-threshold comparisons reported within the same study were retained as separate effect sizes within the corresponding study cluster. Model parameters were estimated using restricted maximum likelihood (REML). Robust variance estimation was not applied. To examine whether the negligible heterogeneity was attributable to the selected model structure, additional sensitivity analyses were conducted using a study-level random-effects model and a conventional random-effects model that treated all effect sizes as statistically independent.

Subgroup analyses and meta-regression analyses were performed to explore potential sources of heterogeneity based on study and outcome characteristics. Prespecified moderators included outcome domain (jump vs. sprint), specific outcome measure (e.g., CMJ, SJ, RSI, 10 m, 15 m, 20 m, and 30 m sprint), VL comparison type (e.g., 10% vs. 20%, 10% vs. 30%, 15% vs. 40%, 20% vs. 40%, or low vs. high VL), participant type, sex, intervention duration, and training frequency. Categorical moderators were examined using subgroup analyses, whereas continuous moderators were examined using meta-regression analyses. Given the limited number of included studies, all subgroup and meta-regression analyses were considered exploratory and were interpreted cautiously.

Heterogeneity was assessed using Cochran’s Q test, τ^2^, and I^2^ statistics, and the proportions of variance attributable to levels 2 and 3 were reported. To assess the robustness of the pooled effect, leave-one-out sensitivity analyses were conducted.

When the number of included studies was sufficient, funnel plots were used to visually assess potential small-study effects, and Egger’s regression test was performed to examine potential publication bias (Egger et al., 1997). All statistical analyses were performed in R. Three-level random-effects meta-analyses, subgroup analyses, and meta-regression analyses were conducted using the metafor package (Viechtbauer, 2010), and figures were generated using ggplot2.

### 3.1 Study selection

A total of 965 records were identified through searches of five electronic databases. Before screening, 102 duplicate records, 93 records removed during preliminary processing, and 34 records removed for other reasons, including conference abstracts, were excluded. Consequently, 736 records remained for title and abstract screening. Following this stage, 391 records were excluded, and 345 reports were sought for retrieval. Of these, 197 reports could not be retrieved, leaving 148 full-text reports for eligibility assessment. During full-text assessment, reports were excluded according to the prespecified operational definitions described in Section 2.3 and **Supplementary Table S3**. Among the reports identified through database searches, 138 were excluded because of a wrong intervention (n = 34), wrong study design (n = 22), wrong outcome (n = 26), insufficient reporting (n = 25), or wrong comparator (n = 31). In addition, 24 records were identified through other methods, including ResearchGate and Google Scholar. Following preliminary screening, 11 records were excluded, and 13 reports were sought for retrieval. Of these, 11 reports were excluded because of a wrong intervention (n = 5), wrong study design (n = 4), or wrong outcome (n = 2), leaving two eligible reports. Each report excluded after full-text assessment was assigned one primary reason for exclusion to avoid double counting. Finally, 10 studies identified through database searches and two studies identified through other methods were included. Thus, a total of 12 randomized controlled trials were included in the systematic review and quantitative synthesis (**Figure 1**; **Supplementary Table S4**) (Galiano et al., 2022; Grazioli et al., 2023a, 2023b; Myrholt et al., 2023; Pareja-Blanco et al., 2017a, 2017b; Pérez-Castilla et al., 2018; Rissanen et al., 2022; Rodríguez-Rosell et al., 2020a, 2021; Rojas-Jaramillo et al., 2024; Wang et al., 2026).

**Figure 1:**
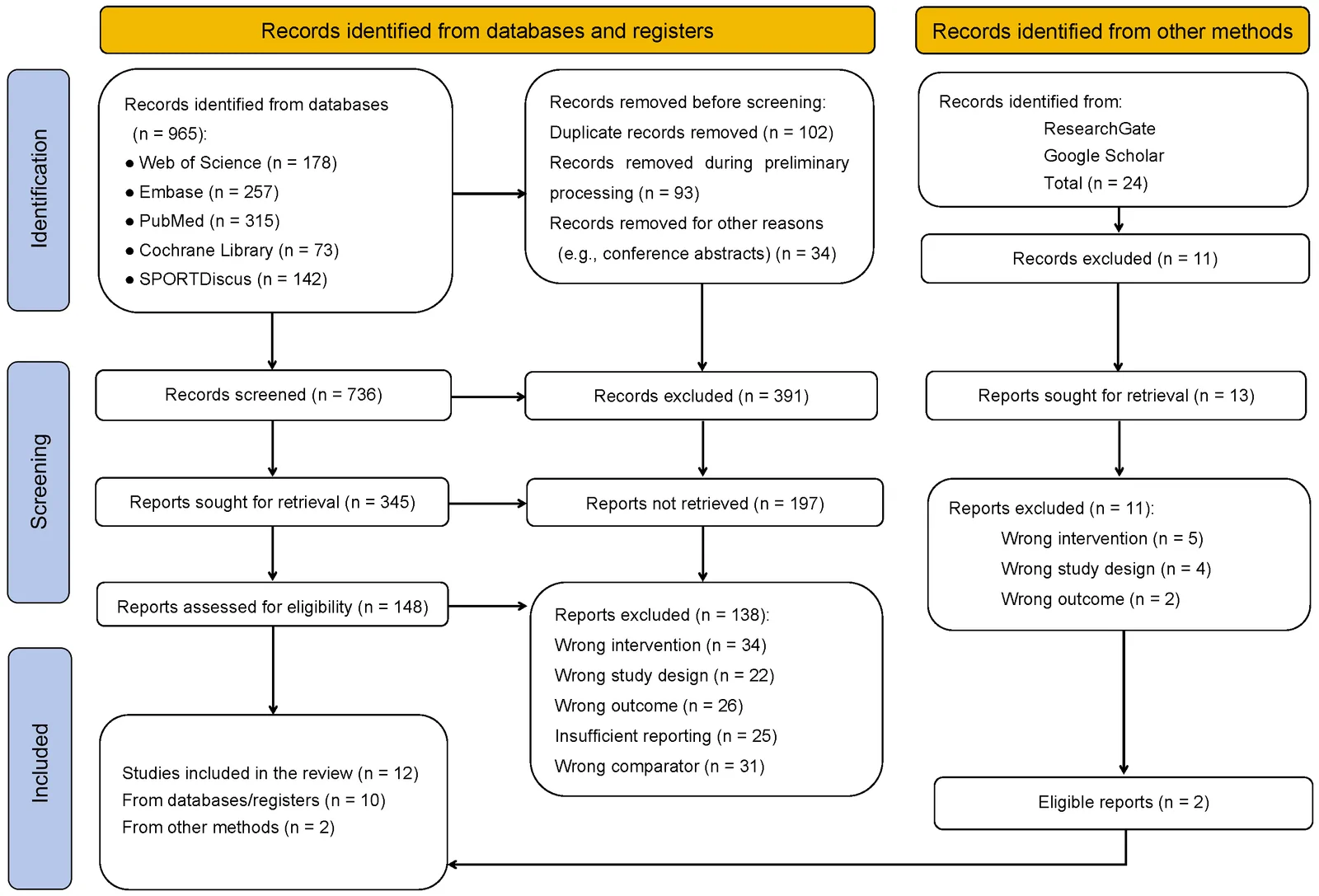
PRISMA 2020 flow diagram of the study identification, screening, eligibility assessment, and inclusion process. Each report excluded after full-text assessment was assigned one primary reason for exclusion according to the prespecified operational definitions described in Section 2.3 and **Supplementary Table S3**, thereby avoiding double counting.

### 3.2 Characteristics of the included studies

Twelve randomized controlled trials published between 2016 and 2026 were included. Participants consisted of youth athletes, professional soccer players, highly trained resistance-trained individuals, and non-athletes with regular resistance training experience. All included samples met the predefined criteria for trained populations. Athlete samples had documented sport-specific training backgrounds, whereas trained non-athlete samples had at least one year of systematic resistance-training experience. Nevertheless, the included populations differed in age, sport background, and training status; therefore, population type was further examined in subgroup analyses. Sample sizes were generally small. The interventions were designed to compare different VL thresholds, including 5% vs. 20%, 10% vs. 20%, 10% vs. 30%, 15% vs. 30%, 15% vs. 40%, 20% vs. 40%, and low VL vs. high VL comparisons. Squat-based training was the most common training mode, while some studies used resisted sled sprint training, loaded countermovement jump training, or combined squat and bench press training. Intervention durations ranged from 4 to 11 weeks, with most studies lasting 4 to 8 weeks, and training frequency was generally 1 to 3 sessions per week. The main outcomes included countermovement jump (CMJ), squat jump (SJ), reactive strength index (RSI), and 10 m, 15 m, 20 m, and 30 m sprint performance. Overall, the included studies showed some clinical and methodological heterogeneity in participant characteristics, VL comparison types, training modes, and outcome measures (**Supplementary Table S4**).

### 3.3 Risk of bias assessment

Risk of bias assessment using RoB 2 showed that all 12 included studies were judged as having “some concerns” overall. Across domains, all studies were rated as “some concerns” for the randomization process (D1), deviations from intended interventions (D2), and selection of the reported result (D5), whereas all studies were rated as “low risk” for measurement of the outcome (D4). For missing outcome data (D3), 5 studies were rated as “low risk” and 7 studies were rated as “some concerns.” Overall, although the included studies generally used relatively objective outcome measures, limitations remained regarding reporting of the randomization process, control of deviations from intended interventions, and prespecification or preregistration of analysis plans. Therefore, the overall risk of bias should be interpreted with caution (**Supplementary Table S6**; **Supplementary Figure S1**).

### 3.4 Meta-analysis results

#### 3.4.1 Overall pooled effect

As shown in **Figure 2**, the three-level random-effects meta-analysis based on 12 studies and 42 effect sizes indicated that lower VL thresholds produced a small but significant favorable effect on combined jump and sprint performance compared with higher VL thresholds (Hedges’ g = 0.29, 95% CI: 0.17 to 0.42, p< 0.001). Most individual effect sizes were distributed to the right of the null-effect line, and the overall pooled effect and its 95% CI also favored lower VL.

**Figure 2:**
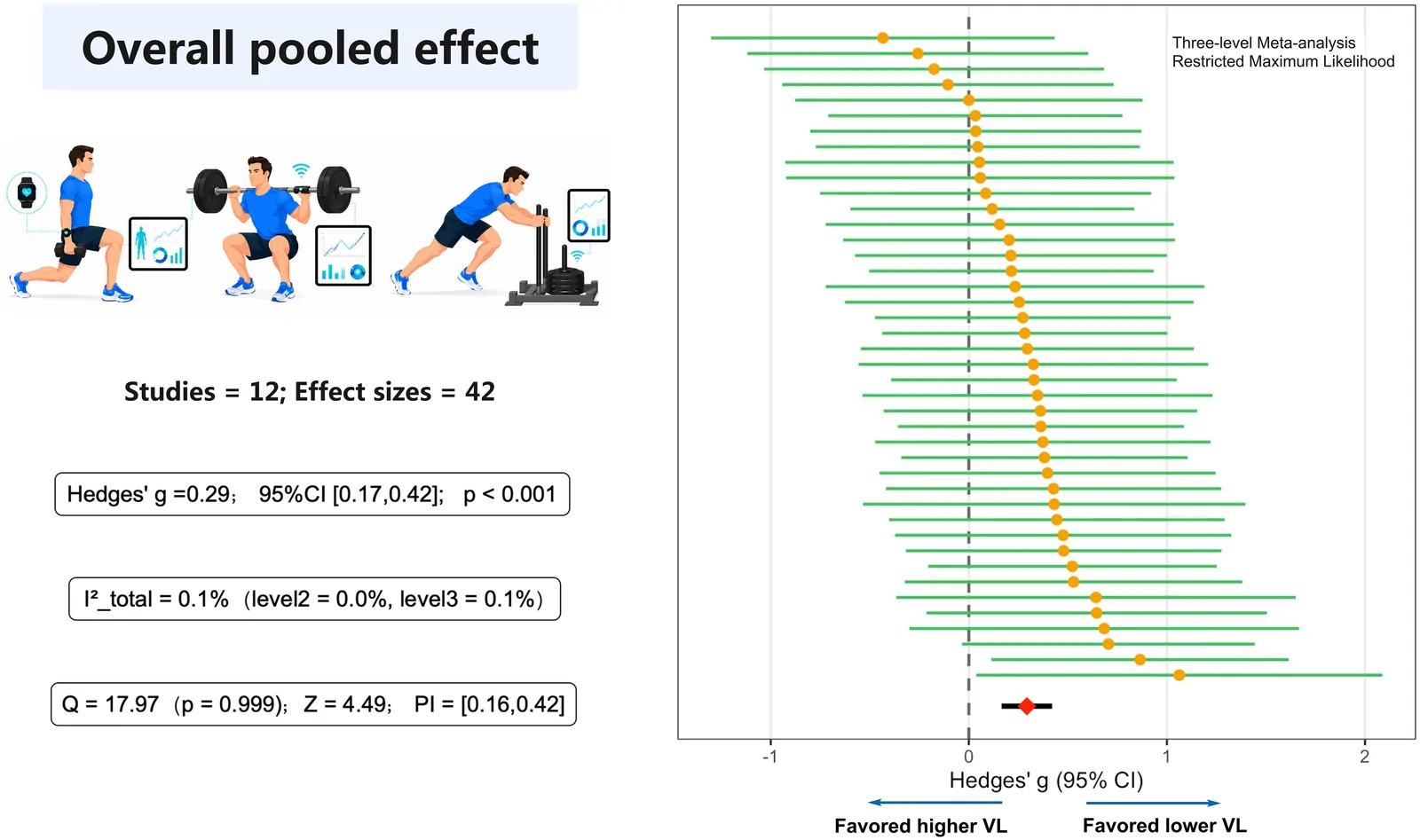
Overall pooled effect of lower versus higher velocity loss thresholds on combined jump and sprint performance. Positive Hedges’ g values favor lower VL thresholds, and the dashed vertical line indicates the null effect. CI, confidence interval; PI, prediction interval; I^2total^, total heterogeneity; Level 2, within-study variance; Level 3, between-study variance; VL, velocity loss.

Residual heterogeneity was very low (I^2^_total = 0.1%), with within-study variance at level 2 accounting for 0.0% and between-study variance at level 3 accounting for 0.1%. Cochran’s Q test did not indicate significant heterogeneity (Q = 17.97, p = 0.999). The overall effect test yielded Z = 4.49, and the prediction interval ranged from 0.16 to 0.42, remaining entirely to the right of the null-effect line. These findings suggest that the direction of evidence was generally consistent.

#### 3.4.2 Effects by outcome domain

To compare the effects of lower versus higher VL thresholds across different outcome domains, effect sizes were categorized into jump performance and sprint performance (**Figure 3**). For jump performance, 22 effect sizes from 12 studies were included, and the pooled effect was significant in favor of lower VL (Hedges’ g = 0.27, 95% CI: 0.10 to 0.45, p = 0.002). For sprint performance, 20 effect sizes from 10 studies were included, and the pooled effect also significantly favored lower VL (Hedges’ g = 0.32, 95% CI: 0.13 to 0.50, p< 0.001).

**Figure 3:**
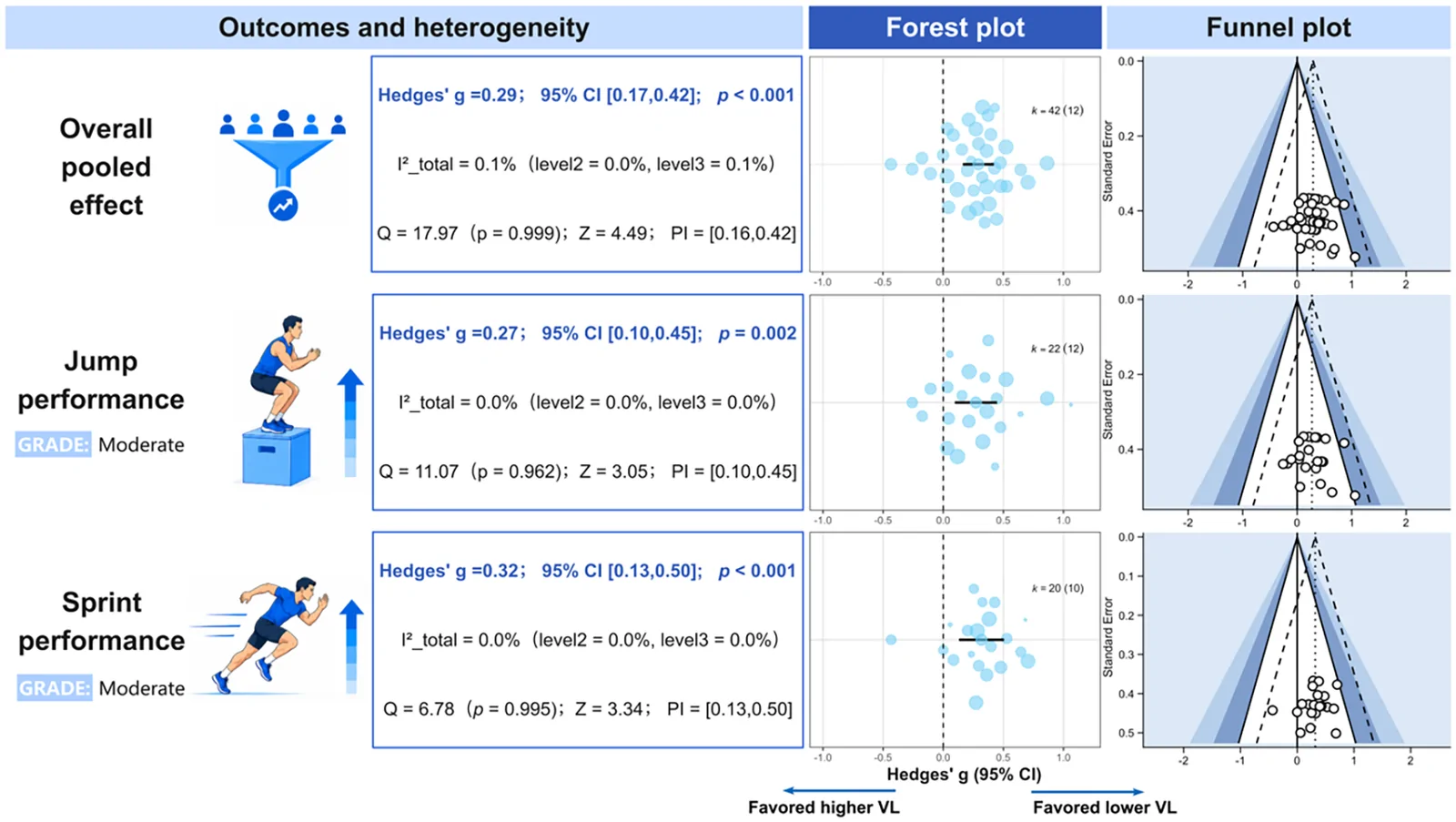
Pooled effects of lower versus higher velocity loss thresholds on jump and sprint performance by outcome domain. Positive Hedges’ g values favor lower VL thresholds, and the dashed vertical line indicates the null effect. CI, confidence interval; PI, prediction interval; I^2total^, total heterogeneity; Level 2, within-study variance; Level 3, between-study variance; GRADE, certainty of evidence; VL, velocity loss.

Residual heterogeneity was low for both jump and sprint outcomes (I^2^_total = 0.0%), with both level 2 and level 3 variance components estimated as 0.0%. Cochran’s Q tests did not indicate significant heterogeneity for either jump performance (Q = 11.07, p = 0.962) or sprint performance (Q = 6.78, p = 0.995). The prediction intervals were 0.10 to 0.45 for jump performance and 0.13 to 0.50 for sprint performance, neither of which crossed the null-effect line. Overall, lower VL showed favorable effects on both jump and sprint performance. Although the pooled effect was numerically larger for sprint performance than for jump performance, both effects were small and should be interpreted cautiously in terms of practical significance (**Supplementary Table S7**).

#### 3.4.3 Effects by specific performance outcome

To further examine the effects of different VL thresholds on specific performance outcomes, separate analyses were conducted for CMJ, SJ, 10 m sprint, 20 m sprint, and 30 m sprint (**Figure 4**). The pooled effect sizes for all specific outcomes were positive, suggesting that lower VL thresholds were generally more favorable than higher VL thresholds for improving jump and sprint performance. However, the magnitude and statistical significance of the effects differed across outcomes.

**Figure 4:**
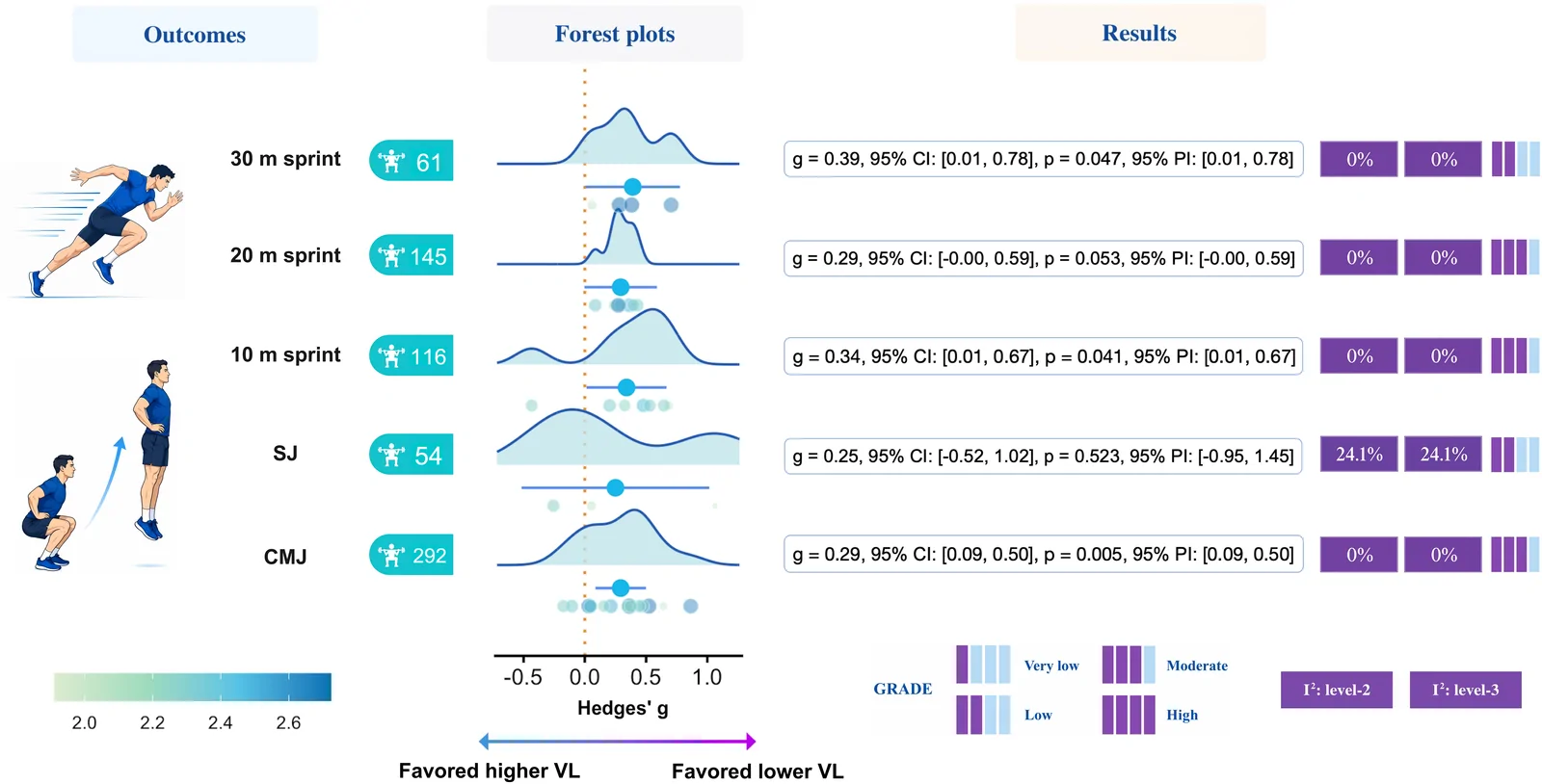
Pooled effects of lower versus higher velocity loss thresholds for specific jump and sprint outcomes. Positive Hedges’ g values favor lower VL thresholds, and the vertical line at 0 indicates the null effect. CI, confidence interval; PI, prediction interval; Level 2, within-study variance; Level 3, between-study variance; GRADE, certainty of evidence; VL, velocity loss.

For jump performance, CMJ showed a significant favorable effect for lower VL (g = 0.29, 95% CI: 0.09 to 0.50, p = 0.005), with moderate certainty of evidence. In contrast, SJ showed a positive but non-significant effect (g = 0.25, 95% CI: −0.52 to 1.02, p = 0.523), with low certainty of evidence.

For sprint performance, significant favorable effects for lower VL were observed for 10 m sprint (g = 0.34, 95% CI: 0.01 to 0.67, p = 0.041) and 30 m sprint (g = 0.39, 95% CI: 0.01 to 0.78, p = 0.047). The certainty of evidence was moderate for 10 m sprint and low for 30 m sprint. The effect for 20 m sprint was also positive but did not reach statistical significance (g = 0.29, 95% CI: −0.00 to 0.59, p = 0.053), with low certainty of evidence.

Overall, lower VL showed clearer favorable effects for CMJ, 10 m sprint, and 30 m sprint, whereas SJ and 20 m sprint showed positive but non-significant trends. These findings suggest that responses to VL thresholds may be outcome-specific. Because evidence for RSI and 15 m sprint was limited and insufficient for stable pooled estimates, these outcomes were not analyzed separately (**Supplementary Table S7**).

#### 3.4.4 Effects by VL comparison type

To further examine whether the type of VL threshold comparison influenced training effects, subgroup analyses were conducted according to VL comparison type (**Figure 5**). Among comparison types with relatively more available data, the 10% vs. 30% VL comparison showed a significant positive effect (g = 0.43, 95% CI: 0.19 to 0.66, p< 0.001), and the 10% vs. 20% VL comparison also reached statistical significance (g = 0.34, 95% CI: 0.06 to 0.62, p = 0.017). In contrast, the pooled effect for the 20% vs. 40% VL comparison was smaller and non-significant (g = 0.15, 95% CI: −0.23 to 0.54, p = 0.430).

**Figure 5:**
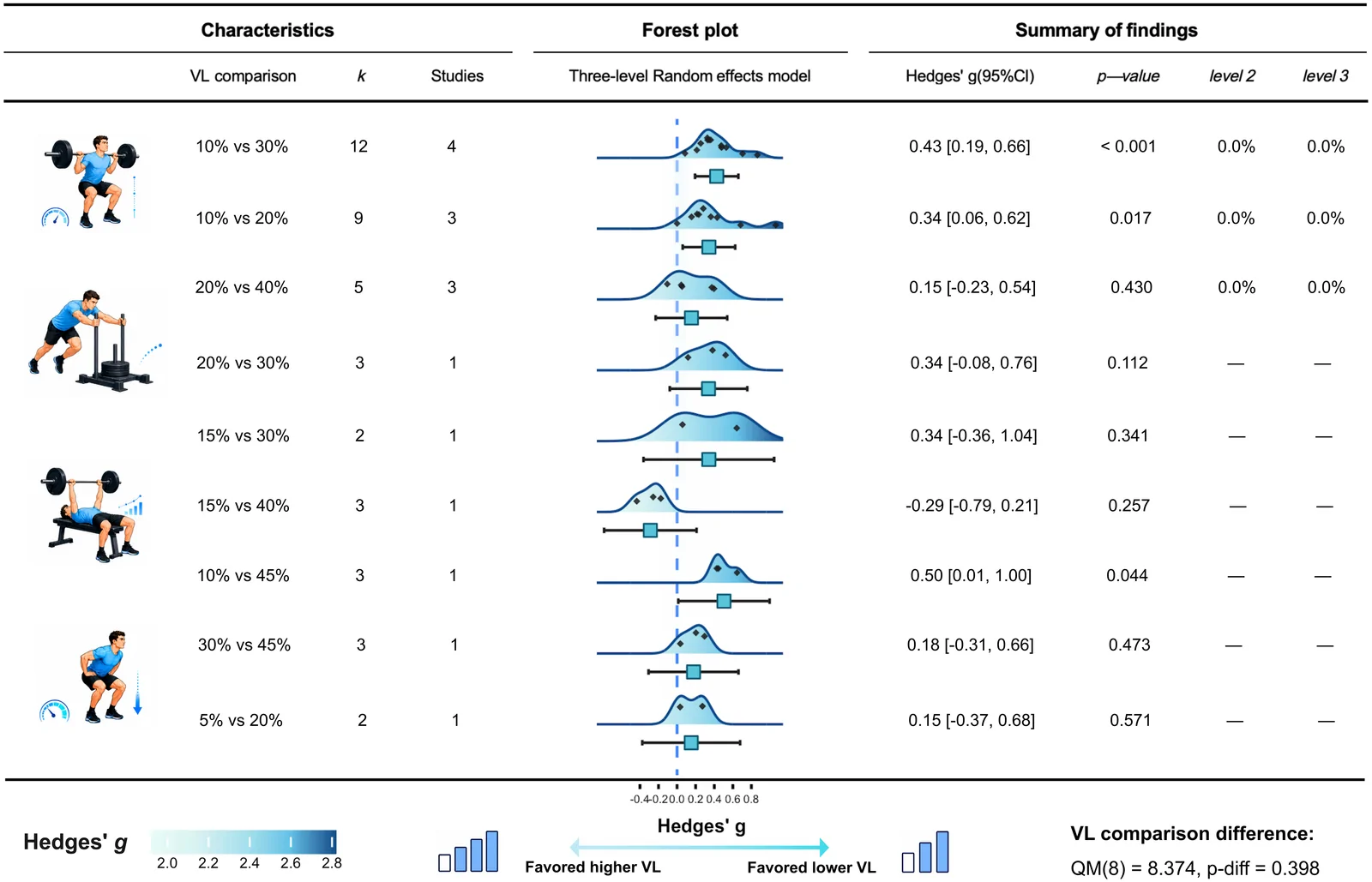
Subgroup effects of lower versus higher velocity loss thresholds by VL comparison type. Ridgeline density curves show the distribution of effect sizes within each subgroup. Squares and horizontal lines indicate the pooled Hedges’ g estimates and corresponding 95% confidence intervals, respectively. The dashed vertical line at g=0 indicates the null effect, and positive values favor lower VL thresholds. p_diff_ indicates the test for differences between subgroups; Level 2 and Level 3 represent within-study and between-study variance, respectively. CI, confidence interval; VL, velocity loss.

Other comparison types, including 20% vs. 30%, 15% vs. 30%, 15% vs. 40%, 10% vs. 45%, 30% vs. 45%, and 5% vs. 20%, were mostly based on data from single studies. Although some comparisons showed positive trends, such as 10% vs. 45% VL (g = 0.50, 95% CI: 0.01 to 1.00, p = 0.044), these findings should be interpreted cautiously because of the limited number of studies and wide confidence intervals.

The overall test for subgroup differences showed no significant difference among VL comparison types (QM(8) = 8.374, p-diff = 0.398), indicating that current evidence is insufficient to demonstrate that any specific VL comparison type is clearly superior to others. Therefore, although lower VL thresholds may show a generally more favorable trend than higher VL thresholds, differences among specific threshold comparisons require further validation in direct comparative studies.

#### 3.4.5 Exploratory moderator and subgroup analyses

To further examine the potential influence of study characteristics and training prescription variables on training effects, moderator analyses were conducted for both categorical and continuous variables. Categorical variables included population type, training frequency, and training mode, and were examined using subgroup analyses within a three-level random-effects model. Continuous variables included intervention duration and VL threshold difference, and were examined using three-level meta-regression analyses to assess their linear associations with effect sizes.

Subgroup analyses for categorical variables showed positive effects in both athletes and trained non-athletes (**Figure 6**). The effect size was g = 0.29 (95% CI: 0.00 to 0.57, p = 0.046) for athletes and g = 0.26 (95% CI: 0.08 to 0.44, p = 0.005) for trained non-athletes, with no significant difference between groups (QM = 0.239, p-diff = 0.625). Regarding training frequency, two sessions per week showed a significant positive effect (g = 0.32, 95% CI: 0.18 to 0.46, p< 0.001), but subgroup differences across training frequencies were not significant (QM = 0.753, p-diff = 0.686). Regarding training mode, squat-based training showed a significant positive effect (g = 0.35, 95% CI: 0.20 to 0.49, p< 0.001), but differences between training modes were also not significant (QM = 2.785, p-diff = 0.426). These findings suggest that the favorable effects of lower VL were generally consistent across population type, training frequency, and training mode, but current evidence is insufficient to conclude that any specific subgroup is superior.

**Figure 6:**
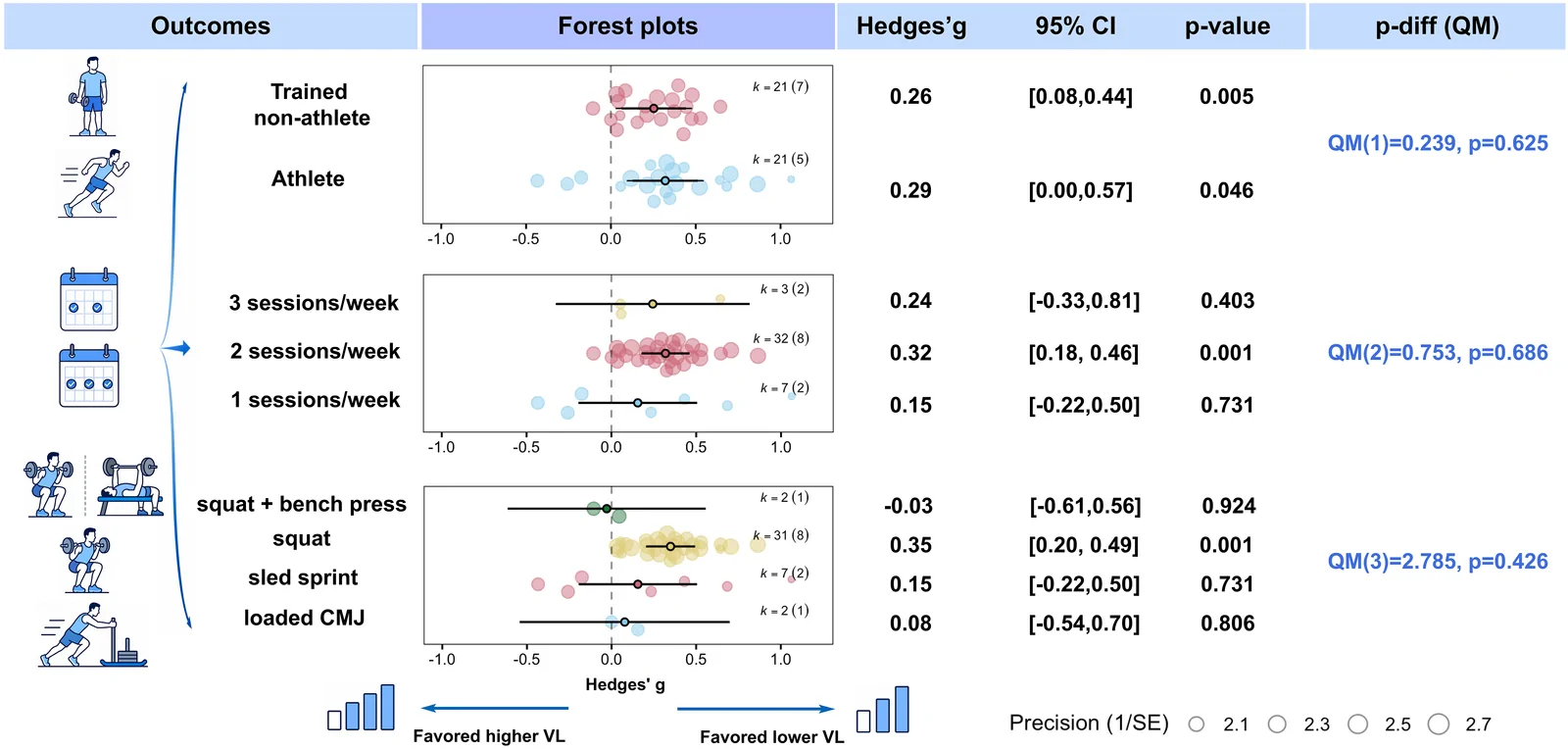
Subgroup effects of lower versus higher velocity loss thresholds by population type, training frequency, and training mode. Positive Hedges’ g values favor lower VL thresholds, and the vertical line at 0 indicates the null effect. Squares and horizontal lines represent pooled effect sizes and corresponding 95% confidence intervals, respectively. p_diff_ indicates the test for differences between subgroups. CI, confidence interval; VL, velocity loss.

Continuous-variable meta-regression analyses showed no significant linear relationship between intervention duration and effect size (β = 0.027, 95% CI: −0.068 to 0.121, p = 0.580), indicating that current evidence does not support a greater advantage of lower VL over higher VL with longer intervention duration (**Figure 7**). VL threshold difference also did not show a significant moderating effect (β = −0.0004, 95% CI: −0.020 to 0.019, p = 0.966), suggesting no clear linear association between the magnitude of the difference between lower and higher VL thresholds and effect size.

**Figure 7:**
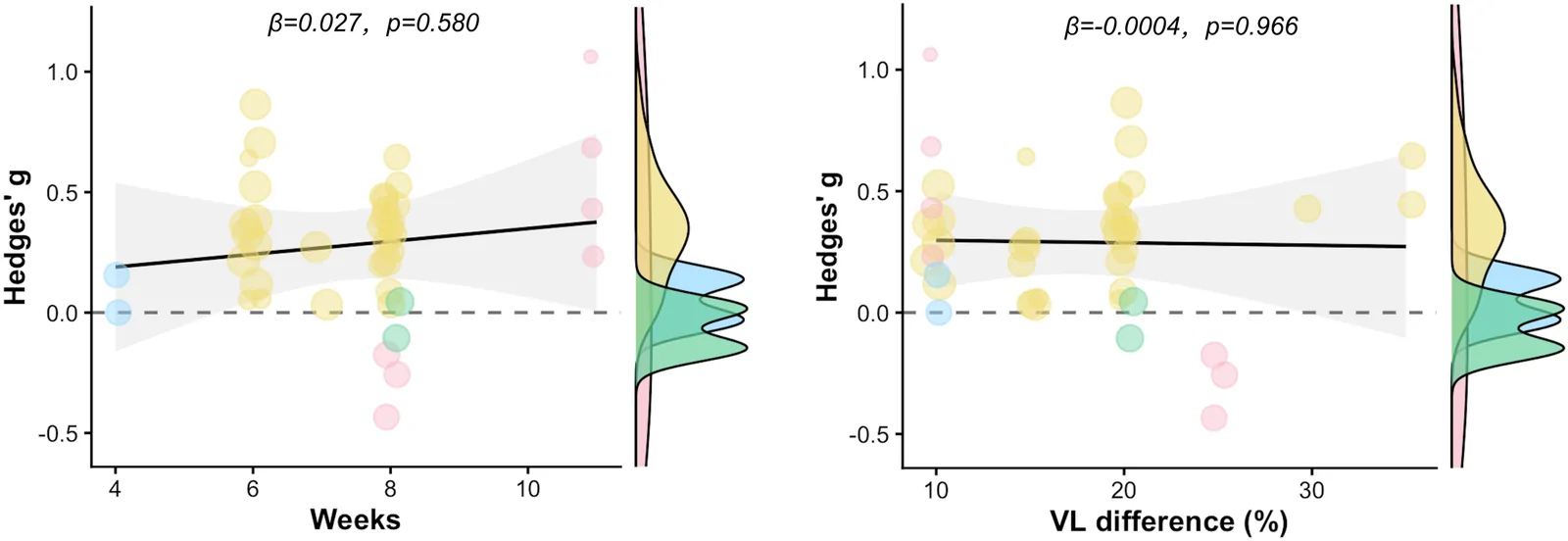
Meta-regression analyses of continuous moderators. The bubble plots illustrate the linear relationships between continuous moderators and effect sizes (Hedges’ g). The solid black line represents the regression line, and the shaded area represents the 95% confidence interval. Bubble size reflects effect size precision. β indicates the regression coefficient, and p indicates the significance level. Different colors represent different training modes: blue = loaded countermovement jump (loaded CMJ), pink = sled sprint, yellow = squat, and green = squat + bench press.

Taken together, the subgroup analyses for categorical variables and meta-regression analyses for continuous variables showed no significant moderating effects of population type, training frequency, training mode, intervention duration, or VL threshold difference. Although some within-subgroup effects reached statistical significance, the tests for between-subgroup differences were not significant. Therefore, these results do not support the conclusion that any specific population, training frequency, training mode, or range of VL threshold difference is superior to others. Given the small number of included studies and the limited number of studies within several subgroups, these moderator findings should be regarded as exploratory rather than confirmatory.

#### 3.4.6 Sensitivity analyses for the assumed pre–post correlation and alternative model specifications

To examine the influence of the assumed pre–post correlation coefficient, effect sizes and sampling variances were recalculated using r = 0.30, r = 0.70, and r = 0.90, in addition to the primary assumption of r=0.50. The pooled effect remained positive and statistically significant under all correlation assumptions, ranging from g=0.25 (95% CI: 0.13 to 0.38) at r= 0.30 to g =0.46 (95% CI: 0.19 to 0.73) at r= 0.90 (all p< 0.001).

Heterogeneity was negligible under the r=0.30 and r=0.50 assumptions (I^2total<^ 0.1%), but increased to 12.7% at r=0.70 and 43.6% at r=0.90. The prediction intervals remained entirely above zero under the r=0.30 and r=0.50 assumptions, whereas they crossed zero under the higher-correlation scenarios of r=0.70 and r=0.90. These findings indicate that the direction and statistical significance of the pooled mean effect were robust to alternative assumptions regarding the pre–post correlation, although the heterogeneity estimates and prediction intervals were more sensitive to higher assumed correlation values (**Supplementary Table S8**).

Additional sensitivity analyses using alternative model specifications yielded virtually identical pooled estimates. The three-level model, the study-level random-effects model, and the conventional random-effects model all produced a pooled effect of g=0.293 (95% CI: 0.166 to 0.421; p<0.001), with negligible heterogeneity across all models. These findings indicate that the direction, magnitude, and statistical significance of the pooled effect were not dependent on the selected random-effects structure (**Supplementary Table S10**).

#### 3.4.7 Leave-one-out sensitivity analysis

To examine the robustness of the overall pooled effect and determine whether any single study had an undue influence on the results, leave-one-out sensitivity analyses were performed. Specifically, one study was removed at a time, and the three-level random-effects model was refitted to assess changes in the pooled effect size, 95% confidence interval, and statistical significance.

The results showed that the pooled effect remained positive after excluding any individual study, with effect sizes ranging from g = 0.247 to 0.334. The overall variation in effect size was small. The 95% confidence intervals after each exclusion did not cross the null-effect line, and all results remained statistically significant (p ≤ 0.001). These findings indicate that the overall pooled effect was not driven by any single study and that no exclusion changed the direction or statistical significance of the results. Overall, the leave-one-out sensitivity analyses suggested that the favorable effect of lower VL thresholds compared with higher VL thresholds on jump and sprint performance in trained populations was robust (**Supplementary Table S9**; **Supplementary Figure S2**).

#### 3.4.8 Publication bias analysis

To assess potential publication bias, power-enhanced funnel plots were first generated at the effect-size level for visual inspection (**Figure 8**). The distributions of effect sizes for overall performance, jump performance, and sprint performance did not show clear unilateral clustering or systematic missingness. Most effect sizes were located in low-power regions, suggesting that some results may have been influenced by small sample sizes or relatively large standard errors. However, no typical pattern of missing studies in high-power regions was observed, and the power-enhanced funnel plots did not reveal a clear visual pattern suggestive of systematic publication bias.

**Figure 8:**
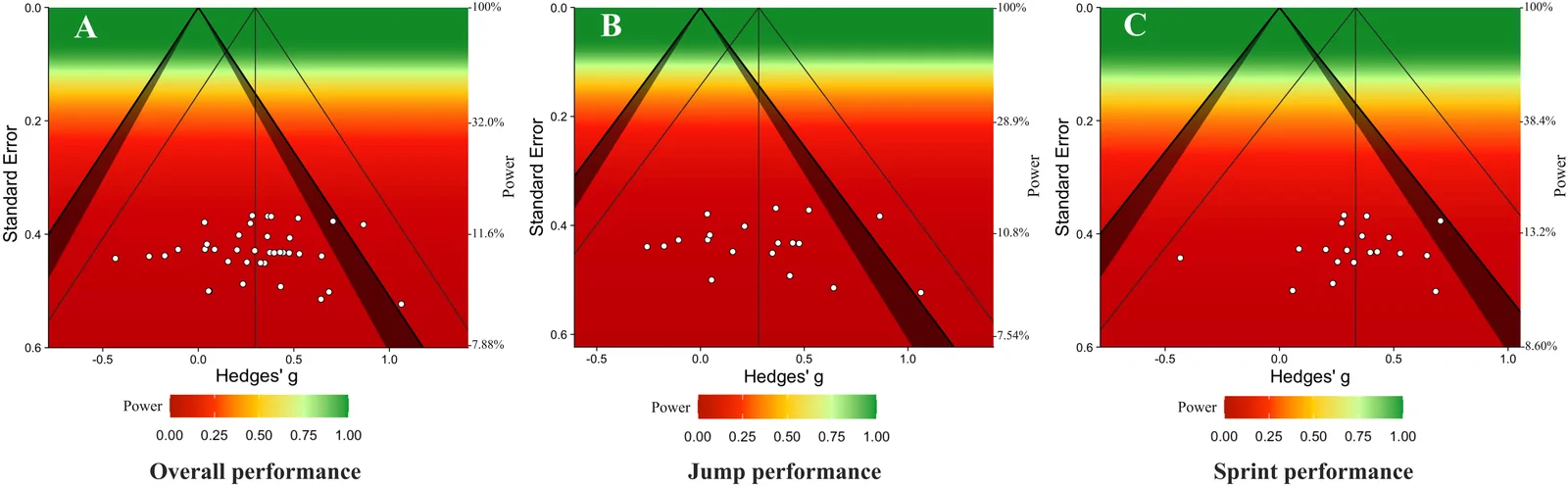
Power-enhanced funnel plots for overall, jump, and sprint performance outcomes. Panels **(A–C)** represent overall, jump, and sprint outcomes, respectively. Each point represents an individual effect size, plotted against its standard error. Background contours indicate increasing statistical power from red to green. Thick funnel lines are centered on the null effect, whereas thin funnel lines are centered on the pooled effect estimate.

Because individual studies could contribute multiple effect sizes, Egger’s test conducted directly at the effect-size level may violate the assumption of independence and underestimate standard errors. Therefore, multiple effect sizes within the same study were further aggregated to the study level. Contour-enhanced funnel plots were then generated using study-level effect sizes, and Egger’s regression tests were performed (**Figure 9**). The results showed that Egger’s tests were not statistically significant for overall performance (p=0.16), jump performance (p=0.61), or sprint performance (p=0.21). Thus, these analyses did not detect statistically significant funnel-plot asymmetry or small-study effects.

**Figure 9:**
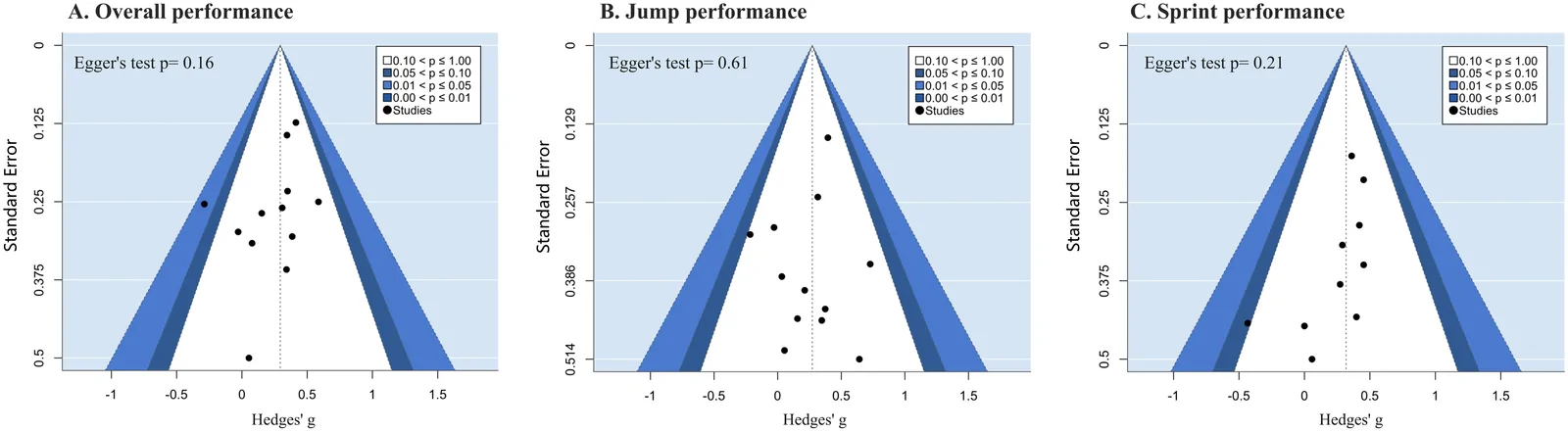
Contour-enhanced funnel plots and Egger’s regression tests for overall, jump, and sprint performance outcomes. Panels **(A–C)** represent overall, jump, and sprint outcomes, respectively. Each point represents an individual effect size plotted against its standard error, and the dashed vertical line indicates the pooled effect estimate. Shaded contour regions correspond to different levels of statistical significance. Egger’s regression test was used to assess potential small-study effects.

Taken together, the power-enhanced funnel plots, contour-enhanced funnel plots, and study-level Egger’s regression tests did not detect clear evidence of systematic publication bias or small-study effects. However, given the limited number of included studies and the presence of several effect sizes in low-power regions, the statistical power of these assessments was limited. Therefore, the possibility of publication bias or small-study effects cannot be excluded, and these findings should be interpreted with caution.

## 4 Discussion

This study systematically evaluated the effects of different velocity loss thresholds during resistance training on jump and sprint performance adaptations in trained populations using a three-level meta-analysis of 12 randomized controlled trials. The results showed that, compared with higher velocity loss thresholds, lower velocity loss thresholds produced a small favorable effect on overall jump and sprint performance, with clearer effects observed for CMJ, 10 m sprint, and 30 m sprint. Although the pooled effect was statistically significant, its magnitude was small, and the effects were not consistent across all outcomes, with nonsignificant findings for SJ and 20 m sprint performance. The certainty of evidence also varied across outcomes. Therefore, lower VL thresholds should not be interpreted as universally superior or optimal across all populations, exercises, and training goals. Exploratory moderator analyses did not detect statistically significant effects of VL comparison type, population type, training frequency, training mode, intervention duration, or VL threshold difference. However, given that only 12 studies were included and that several moderator categories contained relatively few studies, the absence of statistically significant moderator effects should not be interpreted as evidence that these characteristics do not influence training adaptations. Rather, the current evidence is insufficient to determine whether specific training characteristics or threshold differences modify the effects of lower VL. Based on these findings, the following sections discuss the evidence context, potential mechanisms, outcome-specific responses, interpretation of moderator effects, practical applications, and study limitations.

### 4.1 Main findings and evidence context

The core finding of this study was that, compared with higher velocity loss (VL) thresholds, lower VL thresholds during resistance training were more favorable for improving jump and sprint performance in trained populations. The overall pooled effect indicated a small but statistically significant benefit of lower VL, and this effect was observed for both jump and sprint outcomes. In addition, the very low residual heterogeneity, the prediction interval remaining on the favorable side of the null effect, and the stable findings from the leave-one-out sensitivity analysis collectively suggest that the current evidence shows a relatively consistent direction of effect.

This finding is broadly consistent with most previous original studies and systematic reviews (Baena-Marín et al., 2022; Hernández-Belmonte and Pallarés, 2022; Jukic et al., 2023). Existing evidence suggests that lower VL thresholds may be more beneficial for improving jump, sprint, and other speed-oriented performance outcomes, whereas the greater fatigue accumulation associated with higher VL thresholds does not necessarily translate into superior explosive-performance adaptations (Chen et al., 2024; Zhang et al., 2023). Dose–response evidence has also indicated that lower VL ranges may be more suitable for improving CMJ and sprint performance (Liao et al., 2021). However, previous reviews have not reached fully consistent conclusions regarding the relative effects of lower versus higher VL thresholds on athletic performance (Held et al., 2022; Zhang et al., 2023). Compared with previous studies, the present study did not simply examine whether VBT is superior to traditional resistance training. Instead, it directly focused on the comparative effects of different VL thresholds and applied a three-level meta-analytic model to account for the dependency among multiple effect sizes within the same study. This approach is more appropriate for the current evidence structure, in which multiple outcomes and multiple comparisons are commonly reported within individual studies (Assink and Wibbelink, 2016). Therefore, the present study provides more targeted evidence for understanding the relative role of lower VL thresholds in speed–power performance adaptations among trained populations.

### 4.2 Potential explanatory pathways for the effects of lower VL

Several plausible pathways may help explain the small favorable effects of lower velocity loss (VL) thresholds on jump and sprint performance, including neuromuscular recruitment and power production (Cormie et al., 2011; Henneman and Olson, 1965), stretch–shortening cycle (SSC) function (Komi, 1984, 2000), force–velocity specificity (Samozino et al., 2022), and fatigue or effort regulation (Morán-Navarro et al., 2019; Rodríguez-Rosell et al., 2020b, 2018). However, these pathways were not directly examined in the present meta-analysis and should therefore be interpreted as possible explanations rather than confirmed causal mechanisms. One possible interpretation is that the effects of lower VL may relate not only to reduced training volume, but also to the balance among movement quality, technical stability, and fatigue exposure during speed–power-oriented training. Lower VL training typically terminates a set before a marked decline in movement velocity occurs, which may help preserve movement speed, explosive intent, and the proportion of high-quality repetitions (Morán-Navarro et al., 2019; Rodríguez-Rosell et al., 2020b). In contrast, although higher VL training can increase the number of repetitions performed and the total training volume, the later repetitions may progressively deviate from the high-velocity output pattern required for jumping and sprinting as velocity declines and fatigue accumulates (Kons et al., 2023). For example, one study comparing 20% VL with 40% VL during squat training found that the 20% VL group completed fewer total repetitions (185.9 ± 22.2 vs. 310.5 ± 42.0 repetitions) and lower total work (127.5 ± 15.2 vs. 200.6 ± 47.1 kJ), but showed greater improvements in CMJ performance (+9.5% vs. +3.5%) (Pareja-Blanco et al., 2017a). These findings suggest that, for speed–power outcomes such as jumping and sprinting, greater training volume does not necessarily translate into superior sport-specific benefits, and that movement quality and fatigue exposure may represent relevant explanatory factors.

From a neuromuscular adaptation perspective, jumping and sprinting depend heavily on the ability to express force rapidly within a short time frame, including rapid force development, high-threshold motor unit recruitment, intermuscular coordination, and force production under high-velocity conditions (Cormie et al., 2011; Jukic et al., 2023). Under lower VL conditions, athletes can complete more high-quality repetitions at higher movement velocities and under lower levels of fatigue, which may help maintain central neural drive and velocity–power-oriented neuromuscular stimulation (Rodríguez-Rosell et al., 2020a). In addition, CMJ, SJ, and short-distance sprinting all rely on the rapid storage and release of elastic energy within the muscle–tendon system, as well as stable movement rhythm and technical structure (Markovic and Mikulic, 2010; Zhang et al., 2025). Therefore, lower VL training may make the training stimulus more closely aligned with the velocity–power output range required for jumping and sprinting by controlling fatigue, preserving movement velocity, and reducing technical compensations. Conversely, the later repetitions performed under higher VL conditions may reflect greater training volume, metabolic stress, or muscular endurance-type stimulation (Pareja-Blanco et al., 2020; Weakley et al., 2017). Such stimuli may still be valuable for maximal strength, hypertrophy, or the accumulation of training volume, but they may be less specific to jump and sprint performance, for which high-velocity force production is particularly relevant.

### 4.3 Outcome-specific responses in jump and sprint performance

The present findings showed that lower VL thresholds produced favorable effects on both jump and sprint performance. However, the magnitude and statistical significance of the effects were not entirely consistent across outcomes, suggesting that the training effects of VL thresholds may be outcome-specific to some extent (Chen et al., 2024; Jukic et al., 2023). For jump performance, CMJ showed a relatively clear response to lower VL, whereas SJ showed a positive but non-significant trend. This difference may be related to the movement structure of the two tests. CMJ involves a rapid eccentric–concentric transition and relies more heavily on the stretch–shortening cycle (SSC), elastic energy storage and release within the muscle–tendon system, and neuromuscular coordination (Komi, 2000; Van Hooren and Zolotarjova, 2017; Zhang et al., 2025). In contrast, SJ starts from a relatively static semi-squat position, with less contribution from pre-stretch and elastic energy storage, and its performance may be more influenced by initial force production capacity and control of the testing posture (Markovic and Mikulic, 2010). Therefore, by maintaining higher movement velocity, reducing fatigue-related interference, and preserving technical stability, lower VL training may transfer more readily to speed- and coordination-dependent jump tasks such as CMJ. This interpretation is partly supported by one study comparing 10% VL with 30% VL during squat training, which found that, although the 10% VL group completed substantially fewer total repetitions than the 30% VL group (109.6 ± 12.0 vs. 228.0 ± 76.6 repetitions), it showed greater improvements in CMJ performance (+9.2% vs. +5.4%) (Rodríguez-Rosell et al., 2020a).

For sprint performance, both 10 m and 30 m sprint outcomes showed favorable effects of lower VL, whereas the 20 m sprint did not reach statistical significance despite showing the same positive direction. Short-distance sprinting depends strongly on rapid force expression during the acceleration phase, horizontal propulsive ability, and neuromuscular coordination under high-velocity conditions (Morin et al., 2011; Rabita et al., 2015; Samozino et al., 2022). One study reported that the 10% VL group maintained a higher average training velocity than the 30% VL group (0.70 ± 0.01 vs. 0.63 ± 0.01 m·s^-^¹), with more favorable changes in both T10 (−1.6% vs. +0.7%) and T20 (−1.5% vs. +0.4%) (Rodríguez-Rosell et al., 2020a). Another multi-threshold study also showed that, when comparing 10%, 30%, and 45% VL, the 10% VL group demonstrated relatively greater improvements in CMJ and sprint performance, with sprint changes of −2.4%, −1.8%, and −0.5%, respectively (Rodríguez-Rosell et al., 2021). These findings suggest that, as the VL threshold increases, greater training volume or fatigue-related stimulus does not necessarily translate into superior speed–power adaptations. Therefore, the current results may be interpreted as suggesting that the possible favorable effects of lower VL are more apparent in outcomes related to velocity maintenance, SSC utilization, and rapid neuromuscular output, rather than indicating that lower VL consistently improves all jump and sprint outcomes.

### 4.4 Interpretation of moderator effects

The moderator analyses showed that participant type, training frequency, training modality, intervention duration, and the difference between VL thresholds did not significantly moderate the favorable effect of lower VL. Both athletes and trained non-athletes showed favorable responses to lower VL, with no significant between-group difference (QM = 0.239, p = 0.625). The effect size was g = 0.29 (p = 0.046) in athletes and g = 0.26 (p = 0.005) in trained non-athletes. These findings suggest that the positive effect of lower VL may not be limited to a specific type of trained population. However, because the included participants were mainly individuals with previous resistance-training experience, these findings should not be directly generalized to untrained beginners.

Regarding training prescription variables, training frequency, training modality, and intervention duration did not show significant moderating effects. Although training twice per week showed a relatively larger effect size (g = 0.32, p< 0.001), and squat-based training also demonstrated a clear favorable effect (g = 0.35, p< 0.001), the between-subgroup differences were not statistically significant. Therefore, a more conservative interpretation is that, within the context of the currently included studies—interventions lasting 4–11 weeks, mostly involving 1–3 sessions per week, and primarily based on bilateral lower-limb strength exercises—the favorable effect of lower VL appears to be relatively consistent. However, the present evidence is insufficient to conclude that a specific training frequency, intervention duration, or exercise modality is superior.

For the difference between VL thresholds, the meta-regression did not identify a significant linear relationship between the magnitude of the VL difference and effect size (β = −0.0004, p = 0.966). This suggests that the advantage of lower VL may not simply depend on the numerical gap between lower and higher thresholds, but may be more closely related to the ability to maintain higher movement velocity, lower fatigue accumulation, and stable technical output. Similarly, Pérez-Castilla et al. compared 10% VL with 20% VL during power-oriented resistance training and found no significant between-group differences in the force–velocity relationship, mean velocity at different loads, or 15 m sprint performance (Pérez-Castilla et al., 2018). This indicates that, when the difference between VL thresholds is small or when training volume is similar, different low VL thresholds may not necessarily lead to clear performance divergence. Another study also compared lower and higher VL thresholds under similar training-volume conditions and found that both groups improved 1RM by approximately 7%–11%, with no clear between-group differences in jump height or leg-press power; however, the higher VL group showed a slight advantage in vastus lateralis muscle thickness, with a between-group difference of approximately 6 ± 6% (Myrholt et al., 2023). These findings suggest that the effects of VL thresholds may be influenced by training volume and training goals, and that higher VL may still have value for hypertrophy or volume-oriented adaptations. Therefore, lower VL should be viewed as a quality-oriented training regulation strategy rather than a fixed prescription. Its applicability still needs to be further examined under long-term training conditions, different exercise modalities, and controlled training-volume designs.

### 4.5 Practical applications and training implications

The findings of this study provide cautious practical implications for resistance training programs aimed at improving jump and sprint performance. Given the small magnitude of the pooled effect and the low-to-moderate certainty of evidence, lower VL thresholds may be considered as one potential option for maintaining movement velocity, explosive intent, and the proportion of high-quality repetitions rather than as a fixed prescription. During training phases emphasizing CMJ, selected sprint outcomes, and speed–power performance, lower VL may serve as a practical indicator for regulating within-set fatigue. A previous meta-analysis showed that VBT had positive effects on CMJ (SMD = 0.53, p< 0.001) and sprint performance (SMD = −0.40, p< 0.001) in trained individuals, suggesting that velocity-feedback training may have practical value for speed–power development (Zhang et al., 2022). Compared with fixed-repetition prescriptions, VL monitoring allows training volume to be adjusted dynamically according to the athlete’s daily readiness and actual movement velocity, thereby reducing the accumulation of low-quality repetitions after a marked decline in velocity.

From an applied perspective, lower VL may be more suitable during phases emphasizing speed strength, explosive power, and sport-specific transfer, particularly in training contexts that require rapid force production and high-velocity output, such as sprinting, football, basketball, volleyball, and jumping events. A network meta-analysis showed that both low and high VL can improve strength, jump, and sprint performance, but low VL ranked higher for jump performance (Held et al., 2022). A review in elite athletes also suggested that 10%–20% VL may help induce neuromuscular adaptations while reducing neuromuscular fatigue (Włodarczyk et al., 2021). Therefore, in exercises such as squats, jump squats, half squats, and hip–knee extension movements, coaches may use lower VL thresholds as a reference for maintaining output quality and controlling within-set fatigue. When movement velocity clearly declines, technical structure changes, or explosive intent becomes difficult to maintain, terminating the set may be considered.

It should be emphasized that lower VL does not mean that “the lower the threshold, the better”, nor should it be regarded as a fixed optimal prescription for all training goals. From a broader VBT perspective, the meta-analysis by Wang et al. showed that VBT had a small advantage over percentage-based training for jump performance (SMD = 0.27, 95% CI: 0.03 to 0.51), but no significant advantage for maximal strength or sprint performance (Y. Wang et al., 2026). Therefore, VL thresholds should be aligned with the specific training goal. During phases focused on speed–power development, lower VL may be preferred to preserve movement quality. In contrast, during phases targeting basic strength, hypertrophy, or training-volume accumulation, VL thresholds may be moderately increased according to load intensity, training volume, and recovery status (Myrholt et al., 2023). Overall, lower VL should be viewed more as a quality-control tool for jump- and sprint-oriented training than as a rigid prescription to be applied mechanically. Accordingly, VL thresholds should be individualized according to the athlete’s training status, exercise type, training phase, total training volume, and specific performance goals.

### 4.6 Strengths, limitations, and future directions

This study has several methodological and practical strengths. Unlike most previous studies that primarily compared VBT with traditional resistance training (Held et al., 2022; Orange et al., 2022; Zhang et al., 2023), the present study directly focused on the effect differences between different VL thresholds, thereby providing more targeted complementary evidence for understanding the role of VL thresholds in speed–power performance adaptations. In addition, this study used a three-level meta-analytic model to account for the dependency among multiple effect sizes within the same study. Compared with traditional two-level models, this approach is more appropriate for the current VL literature, in which multiple outcomes and multiple comparisons are often reported within individual studies (Assink and Wibbelink, 2016). Furthermore, this study distinguished between jump and sprint performance and further analyzed specific outcomes, including CMJ, SJ, 10 m, 20 m, and 30 m sprint performance, which may help clarify the outcome-specific effects of lower VL.

Several limitations should also be acknowledged. First, the moderator analyses were based on only 12 included studies, and several subgroup categories, such as SJ, 20 m sprint, and specific VL comparison forms, contained relatively few studies or effect sizes. Consequently, these analyses had limited statistical power and should be interpreted as exploratory rather than confirmatory, with the possibility of false-negative moderator findings. Accordingly, the absence of statistically significant moderator effects should not be interpreted as evidence that the examined population or training characteristics do not influence training adaptations. Second, the included studies differed in VL threshold settings, exercise selection, load intensity, measurement devices, measurement positions, and performance testing methods, which may have influenced the stability of the effect estimates. Third, the current evidence was mainly derived from individuals with previous resistance-training experience. Therefore, the applicability of these findings to women, youth athletes, beginners, and athletes from different sports requires further investigation. In addition, the intervention duration of the included studies ranged from 4 to 11 weeks, with most lasting 4 to 8 weeks, leaving the long-term effects of different VL thresholds within periodized training programs unclear. Finally, evidence for outcomes such as RSI, 15 m sprint, change-of-direction ability, and repeated-sprint ability remains limited, which restricts a more comprehensive interpretation of different dimensions of speed–power performance.

Future studies should conduct larger and more rigorously designed randomized controlled trials to further examine the dose–response relationship between different VL thresholds and jump and sprint performance. In particular, studies with controlled training volume are needed to distinguish the independent effects of VL thresholds from those of total training volume, while improving methodological rigor in randomization, allocation concealment, preregistration, and outcome reporting (Schulz et al., 2010). Future research should also extend to populations differing in sex, age, training status, and sport specialization, and incorporate measures such as surface electromyography, tendon mechanics, three-dimensional motion analysis, and fatigue monitoring to clarify the mechanisms through which lower VL affects different performance outcomes. Overall, the current evidence supports lower VL as a quality-control strategy in speed–power training, but its long-term effects, boundaries of application, and individualized regulation strategies require further investigation.

## 5 Conclusions

Based on 12 randomized controlled trials and 42 effect sizes, lower VL resistance training produced a small favorable effect on combined jump and sprint performance compared with higher VL thresholds in trained populations. Evidence was clearer for CMJ, 10 m sprint, and 30 m sprint performance, whereas findings for SJ and 20 m sprint performance were less certain. Lower VL may therefore represent a potentially useful option for speed–power-oriented training, but the present findings do not establish it as universally superior or optimal across all populations, exercises, and training goals. The small favorable effects may be partly related to the maintenance of movement velocity, reduced within-set fatigue accumulation, a higher proportion of high-quality repetitions, and greater specificity to speed–power performance; however, these potential mechanisms were not directly examined in the present meta-analysis. Practical application should therefore be individualized according to the training objective, exercise type, training phase, total training volume, and athlete response. Further high-quality randomized controlled trials are needed to clarify the dose–response relationship and the contexts in which different VL thresholds are most appropriate.

## Data Availability

The original contributions presented in the study are included in the article/**Supplementary Material**. Further inquiries can be directed to the corresponding author/s.
