# Supplementary material for: Effects of different velocity loss thresholds during resistance training on jump and sprint performance in trained populations: a systematic review and three-level meta-analysis
Source: Front Physiol. 2026 Oct 6;17:1893020. doi: 10.3389/fphys.2026.1893020 (PMC13680193; doi:10.3389/fphys.2026.1893020)
Supplement: Supplementary file 1 [file DataSheet1.pdf]

ELECTRONIC SUPPLEMENTARY MATERIAL CONTENTS

Number	Material	Page
1	Electronic Supplementary Material Appendix S1 (PRISMA 2020 checklist)	1-3
2	Electronic Supplementary Material Appendix S2 (Search strategy)	4
3	Electronic Supplementary Material Appendix S3<br>(Inclusion and exclusion criteria and operational definitions of full-text exclusion categories.)	5-6
4	Electronic Supplementary Material Appendix S4 (Characteristics of the included studies)	7-10
5	Electronic Supplementary Material Appendix S5 (Details of numerical data extracted from graphical presentation)	11
6	Electronic Supplementary Material Appendix S6 (Risk of Bias in Included Studies)	11-12
7	Electronic Supplementary Material Appendix S7 (GRADE evidence profile)	13
8	Electronic Supplementary Material Appendix S8<br>(Sensitivity analyses using alternative assumed pre–post correlation coefficients)	14
9	Electronic Supplementary Material Appendix S9 (Leave-one-out analysis)	15
10	Electronic Supplementary Material Appendix S10<br>(Sensitivity analyses using alternative model specifications)	16

Electronic Supplementary Material Appendix S1 (PRISMA 2020 checklist)

Table S1. PRISMA checklist

Section and Topic	Item	Checklist item	Reported on page #
Effects of Different Velocity Loss Thresholds During Resistance Training on Jump and Sprint Performance in Trained Populations: A Systematic Review and Three-Level Meta-Analysis			
Title	1	Identify the report as a systematic review, meta-analysis, or both.	
**ABSTRACT**			
Abstract	2	Provide a structured summary including, as applicable: background; objectives; data sources; study eligibility criteria, participants, and interventi ons; study appraisal and synthesis methods; results; limitations; conclusions and implications of key findings; systematic review registration nu mber.	1
**INTRODUCTION**			
Rationale	3	Describe the rationale for the review in the context of existing knowledge.	1，2
Objectives	4	Provide an explicit statement of questions being addressed with reference to participants, interventions, comparisons, outcomes, and study design （PICOS）.	3，4
**METHODS**			
Protocol and registration	5	Indicate if a review protocol exists, if and where it can be accessed (e.g., Web address), and, if available, provide registration information inclu ding registration number.	3
Eligibility criteria	6	Specify study characteristics (e.g., PICOS, length of follow-up) and report characteristics (e.g., years considered, language, publication status) u sed as criteria for eligibility, giving rationale.	3，4
Information sources	7	Describe all information sources (e.g., databases with dates of coverage, contact with study authors to identify additional studies) in the search and date last searched.	3
Search	8	Present full electronic search strategy for at least one database, including any limits used, such that it could be repeated.	3
Study selection	9	State the process for selecting studies (i.e., screening, eligibility, included in systematic review, and, if applicable, included in the meta-analysi	3，7

Section and Topic	Item	Checklist item	Reported on page #
		s).	
Data collection process	10	Describe all information sources (e.g., databases with dates of coverage, contact with study authors to identify additional studies) in the search and date last searched.	3，4
Data items	11	List and define all variables for which data were sought (e.g., PICOS, funding sources) and any assumptions and simplifications made.	3，4
Risk of bias in individual studies	12	Describe methods used for assessing risk of bias of individual studies (including specification of whether this was done at the study or outcom e level), and how this information is to be used in any data synthesis.	3，4
Summary measures	13	State the principal summary measures (e.g., risk ratio, difference in means).	3，4
Synthesis of results	14	Describe the methods of handling data and combining results of studies, if done, including measures of consistency (e.g., I^2) for each meta-anal ysis.	3，4
Risk of bias across studies	15	Specify any assessment of risk of bias that may affect the cumulative evidence (e.g., publication bias, selective reporting within studies).	3，4
Additional analyses	16	Describe methods of additional analyses (e.g., sensitivity or subgroup analyses, meta-regression), if done, indicating which were pre-specified.	4
**RESULTS**			
Study selection	17	Give numbers of studies screened, assessed for eligibility, and included in the review, with reasons for exclusions at each stage, ideally with a flow diagram.	5，6
Study characteristics	18	For each study, present characteristics for which data were extracted (e.g., study size, PICOS, follow-up period) and provide the citations.	5，6
Risk of bias in studies	19	Present data on risk of bias of each study and, if available, any outcome level assessment (see item 12）。	6，7
Results of individual	20	For all outcomes considered (benefits or harms), present, for each study: (a) simple summary data for each intervention	8，9，10

Section and Topic	Item	Checklist item	Reported on page #
studies		group (b) effect estimates and confidence intervals, ideally with a forest plot.	
Synthesis of results	21	Present results of each meta-analysis done, including confidence intervals and measures of consistency.	10，11，12
Risk of bias across studies	22	Present results of any assessment of risk of bias across studies (see Item 15).	12，13，14
Additional analysis	23	Give results of additional analyses, if done (e.g., sensitivity or subgroup analyses, meta-regression [see Item 16]).	13，14
**DISCUSSION**			
Summary of evidence	24	Summarize the main findings including the strength of evidence for each main outcome; consider their relevance to key groups (e.g., healthcare providers, users, and policy makers).	14，15，16
Limitations	25	Discuss limitations at study and outcome level (e.g., risk of bias), and at review-level (e.g., incomplete retrieval of identified research, reporting bias).	17，18，19
Conclusions	26	Provide a general interpretation of the results in the context of other evidence, and implications for future research.	19
FUNDING			
Funding	27	Describe sources of funding for the systematic review and other support (e.g., supply of data); role of funders for the systematic review.	n/a

Electronic Supplementary Material Appendix S2 (Search strategy)

Table S2. Search strategy

Database	retrieval strategy
WOS	TS=(("velocity loss" OR "velocity-loss" OR "velocity loss threshold" OR "velocity-loss threshold" OR "velocity based training" OR "velocity-based training" OR "VBT") AND ("resistance training" OR "strength training" OR "resistance exercise" OR "weight training" OR "strength exercise") AND (jump OR jumping OR "jump performance" OR "countermovement jump" OR "CMJ" OR "squat jump" OR "SJ" OR sprint OR sprinting OR "sprint performance" OR "linear sprint" OR "running speed") AND (trained OR "trained individuals" OR athlete OR "resistance-trained" OR "strength-trained"))
Embase	('velocity loss':ti,ab,kw OR 'velocity-loss':ti,ab,kw OR 'velocity loss threshold':ti,ab,kw OR 'velocity-loss threshold':ti,ab,kw OR 'velocity based training':ti,ab,kw OR 'velocity-based training':ti,ab,kw OR vbt:ti,ab,kw) AND ('resistance training':ti,ab,kw OR 'strength training':ti,ab,kw OR 'resistance exercise':ti,ab,kw OR 'weight training':ti,ab,kw OR 'strength exercise':ti,ab,kw) AND (jump:ti,ab,kw OR jumping:ti,ab,kw OR 'jump performance':ti,ab,kw OR 'countermovement jump':ti,ab,kw OR cmj:ti,ab,kw OR 'squat jump':ti,ab,kw OR sj:ti,ab,kw OR sprint:ti,ab,kw OR sprinting:ti,ab,kw OR 'sprint performance':ti,ab,kw OR 'linear sprint':ti,ab,kw OR 'running speed':ti,ab,kw) AND (trained:ti,ab,kw OR 'trained individuals':ti,ab,kw OR athlete:ti,ab,kw OR 'resistance-trained':ti,ab,kw OR 'strength-trained':ti,ab,kw)
Pubmed	#1 "Velocity Loss"[Title/Abstract] OR "Velocity-Loss"[Title/Abstract] OR "Velocity Loss Threshold"[Title/Abstract] OR "Velocity Loss Thresholds"[Title/Abstract] OR "Velocity-Loss Threshold"[Title/Abstract] OR "Velocity-Loss Thresholds"[Title/Abstract] OR "Velocity Based Training"[Title/Abstract] OR "Velocity-Based Training"[Title/Abstract] OR "VBT"[Title/Abstract]<br>#2 "Resistance Training"[Mesh] OR "Resistance Training"[Title/Abstract] OR "Strength Training"[Title/Abstract] OR "Resistance Exercise"[Title/Abstract] OR "Resistance Exercises"[Title/Abstract] OR "Weight Training"[Title/Abstract] OR "Strength Exercise"[Title/Abstract] OR "Strength Exercises"[Title/Abstract]<br>#3 "Jumping"[Mesh] OR jump[Title/Abstract] OR jumping[Title/Abstract] OR "Jump Performance"[Title/Abstract] OR "Countermovement Jump"[Title/Abstract] OR "CMJ"[Title/Abstract] OR "Squat Jump"[Title/Abstract] OR "SJ"[Title/Abstract] OR sprint[Title/Abstract] OR sprinting[Title/Abstract] OR "Sprint Performance"[Title/Abstract] OR "Linear Sprint"[Title/Abstract] OR "Running Speed"[Title/Abstract]<br>#4 trained[Title/Abstract] OR "trained individuals"[Title/Abstract] OR athlete[Title/Abstract] OR athletes[Title/Abstract] OR "resistance-trained"[Title/Abstract] OR "strength-trained"[Title/Abstract] OR "trained men"[Title/Abstract] OR "trained women"[Title/Abstract]<br>#5 #1 AND #2 AND #3 AND #4
Cochrane Library	#1 "velocity loss" OR "velocity-loss" OR "velocity loss threshold" OR "velocity loss thresholds" OR "velocity-loss threshold" OR "velocity-loss thresholds" OR "velocity based training" OR "velocity-based training" OR "VBT"<br>#2 "resistance training" OR "strength training" OR "resistance exercise" OR "resistance exercises" OR "weight training" OR "strength exercise" OR "strength exercises"<br>#3 jump OR jumping OR "jump performance" OR "countermovement jump" OR "CMJ" OR "squat jump" OR "SJ" OR sprint OR sprinting OR "sprint performance" OR "linear sprint" OR "running speed"<br>#4 #1 AND #2 AND #3
SPORTDiscus	TX ("velocity loss" OR "velocity-loss" OR "velocity loss threshold" OR "velocity loss thresholds" OR "velocity-loss threshold" OR "velocity-loss thresholds" OR "velocity based training" OR "velocity-based training" OR "VBT") AND TX ("resistance training" OR "strength training" OR "resistance exercise" OR "resistance exercises" OR "weight training" OR "strength exercise" OR "strength exercises") AND TX (jump OR jumping OR "jump performance" OR "countermovement jump" OR "CMJ" OR "squat jump" OR "SJ" OR sprint OR sprinting OR "sprint performance" OR "linear sprint" OR "running speed")

Electronic Supplementary Material Appendix S3

(Inclusion and exclusion criteria and operational definitions of full-text exclusion categories)

Table S3. Inclusion and exclusion criteria and operational definitions of full-text exclusion categories.

Inclusion and Exclusion Criteria
**Inclusion criteria**
(1) Participants: Participants were competitive athletes with a documented sport-specific training background or non-athletes with at least one year of regular resistance-training experience. Youth participants were eligible only when a clearly defined sport-specific and/or resistance-training background was reported. No restrictions were imposed on age or sex, provided that the above training-background criterion was met.
(2) Intervention: The intervention involved resistance training in which an explicitly defined velocity loss (VL) threshold was used as the primary criterion for regulating repetition volume and/or terminating a set. Studies were required to compare lower and higher VL thresholds.
(3) Comparator: The comparator had to involve comparisons between different VL thresholds, such as 10% vs. 20%, 10% vs. 30%, 15% vs. 30%, 15% vs. 40%, 20% vs. 40%, or low VL vs. high VL. Studies with two or more clearly defined VL-threshold groups were eligible. If a study included additional comparator conditions, only data permitting a direct comparison between lower and higher VL thresholds were extracted.
(4) Outcomes: Studies were required to report at least one outcome related to jump or sprint performance. Jump outcomes included, but were not limited to, countermovement jump (CMJ), squat jump (SJ), reactive strength index (RSI), and other vertical jump performance measures. Sprint outcomes included, but were not limited to, 10 m, 15 m, 20 m, and 30 m sprint performance, as well as other linear sprint performance measures. Studies had to provide sufficient data for extracting or calculating effect sizes, such as sample size, pre- and post-intervention means and standard deviations, change scores and corresponding dispersion measures, or other data that could be converted into effect sizes.
(5) Study design: Randomized controlled trials (RCTs) were included. Studies had to use a longitudinal training intervention design and include at least two training groups with different VL thresholds to compare the effects of lower versus higher VL on jump or sprint performance.
**Exclusion criteria**
(1) Studies were excluded if participants were untrained general populations, individuals without regular training experience, clinical or rehabilitation populations, or child/adolescent samples without a clearly defined training background.<br>(2) Studies were excluded if VL was not used as the core regulatory variable. This included studies using only traditional percentage-based loading, studies using velocity feedback without clearly defined VL thresholds, studies comparing VBT with percentage-based training (PBT) without extractable data for comparisons between different VL thresholds, and interventions that did not fall within the scope of resistance training.
(3) Studies were excluded if they did not directly compare two or more different VL thresholds, or if the VL groups were not clearly defined. Studies reporting only one VL-threshold training group without another eligible VL-threshold comparison group were excluded from the review and meta-analysis.
(4) Studies were excluded if they did not report jump- or sprint-related outcomes. This included studies reporting only maximal strength, hypertrophy, power output, metabolic responses, perceived fatigue, or technical variables without jump or sprint performance data. Studies from which the data required to extract or calculate effect sizes could not be obtained or converted were also excluded.
(5) Reviews, meta-analyses, conference abstracts, dissertations, book chapters, commentaries, expert opinions, methodological papers, and

animal studies were excluded. Cross-sectional studies, acute experimental studies, observational studies without a training intervention, and studies that did not report pre- and post-intervention results were also excluded.

Operational definitions of full-text exclusion categories

Wrong intervention	Resistance training was not regulated by an explicitly defined velocity loss (VL) threshold, or the intervention was outside the scope of longitudinal resistance training.
Wrong comparator	The study did not provide a direct comparison between two or more different VL thresholds.
Wrong outcome	The study did not report an eligible jump or linear sprint performance outcome.
Insufficient reporting	The data required to calculate an effect size were unavailable and could not be derived from the article, tables, figures, or supplementary materials.
Wrong study design	The study was not a randomized longitudinal training trial, including acute, cross-sectional, observational, or non-original research designs.

Electronic Supplementary Material Appendix S4
(Characteristics of the included studies)

Table S4. Characteristics of the included studies

Study	Participant characteristics	Sample size (N)	Population status	Training background	VL threshold comparison	Intervention duration / frequency	Training protocol	Main outcomes
Wang et al. 2026	VL10% : 16.87 ± 0.83 years, 180.47 ± 4.64 cm, 67.97 ± 4.97 kg; VL20% : 17.07 ± 0.79 years, 179.60 ± 4.95 cm, 67.03 ± 5.69 kg; VL30% : 16.93 ± 0.96 years, 179.87 ± 4.32 cm, 67.23 ± 5.13 kg.	VL10：N=15 VL20：N=15 VL30：N=15	Youth sprinters	Sprint athletes; ≥2 years of resistance training; ≥3 years of sprint-specific training; proficient in squatting.	10%VL vs 20%VL vs 30%VL	6 weeks; 2 sessions/week.	Barbell back squat VBT at 80% 1RM; 20 total repetitions/set; 2-min inter-set rest; repetitions controlled by VL thresholds.	CMJ、RSI 30m sprint
Rojas-Jaramillo et al. 2024	VL10%: 16.3 ± 0.2 years, 73.3 ± 4.7 kg, 1.73 ± 0.05 m; VL30%: 16.6 ± 0.3 years, 73.6 ± 4.3 kg, 1.72 ± 0.06 m.	VL10：N=10 VL30：N=10	Youth soccer players	Soccer players; >5 years of training experience; injury-free in the past 3 months.	10%VL vs 30%VL	8 weeks; 2 sessions/week.	Full squat training only; 45–60% 1RM; repetitions controlled by VL thresholds; 3-min inter-set rest.	CMJ 10m sprint 20m sprint
Pareja-Blanco et al. 2016	Age: 23.8 ± 3.4/3.5 years Height: 1.74 ± 0.07 m Body mass: 75.5 ± 8.6 kg	VL15：N=8 VL30：N=8	Competitive athletes (professional soccer players)	Resistance-trained; proficient in the full squat exercise.	15%VL vs 30%VL	6 weeks; 3 sessions/week.	Full squat training only; 50–70% 1RM; repetitions controlled by VL thresholds; 4-min inter-set rest.	CMJ 30m sprint
Grazioli et al. 2023	VL15% : 25.5 ± 6.0 years, 181.5 ± 9.0 cm, 82.2 ± 9.6 kg; VL40% : 26.3 ± 5.1 years, 176.6 ± 7.1 cm, 76.0 ± 9.2 kg.	VL15：N=11 VL40：N=10	Competitive athletes (high-level professional soccer players)	High-level soccer players; competed at national/state level.	15%VL vs 40%VL	8 weeks; 9 sessions in total (2 sessions in week 1 and 1 session/week for the following 7 weeks).	Resisted sled sprint training; load prescribed by VL thresholds; 30-s rest between sprints; performed on artificial turf.	CMJ、SJ 10m sprint

Grazioli et al. 2020	VL10% : 26.25 ± 5.31 years, 178.62 ± 9.48 cm, 75.76 ± 9.76 kg; VL20% : 25.44 ± 3.57 years, 181.22 ± 8.19 cm, 79.45 ± 9.94 kg.	VL10：N=8 VL20：N=9	Competitive athletes (professional soccer players)	Professional soccer players; weekly training and matches; injury-free.	10%VL vs 20%VL	11 weeks; 1 session/week.	20-m resisted sled sprint training; load at 45–65% body mass; 30-s rest between repetitions; set termination controlled by VL thresholds.	CMJ、SJ 10m sprint 20m sprint
Rodrí guez-Rosell et al. 2020	VL10%: 22.8 ± 3.1 years, 75.1 ± 10.3 kg, 1.77 ± 0.08 m; VL30%: 22.2 ± 2.7 years, 74.0 ± 9.1 kg, 1.76 ± 0.07 m.	VL10：N=12 VL30：N=13	Non-athletes (healthy trained individuals)	Physical education students; 1–3 years of resistance training; injury-free in the past 6 months.	10%VL vs 30%VL	8 weeks; 2 sessions/week.	Full squat training only; 70–85% 1RM; repetitions controlled by VL thresholds; 4-min inter-set rest.	CMJ 10m sprint 20m sprint
Rodríguez-Rosell et al. 2021	VL10%: 22.8 ± 3.9 years, 70.7 ± 5.1 kg, 1.76 ± 0.04 m; VL30%: 21.9 ± 2.3 years, 73.7 ± 9.4 kg, 1.76 ± 0.07 m; VL45%: 21.6 ± 2.8 years, 72.1 ± 9.6 kg, 1.72 ± 0.08 m.	VL10：N=11 VL30：N=11 VL45：N=11	Non-athletes (healthy trained individuals)	Physical education students; 1–3 years of resistance training; injury-free in the past 6 months.	10%VL vs 30%VL vs 45%VL	8 weeks; 2 sessions/week.	Full squat training only; 55–70% 1RM; 3 sets/session; repetitions controlled by VL thresholds; 4-min inter-set rest.	CMJ 10m sprint 20m sprint
Pareja-Blanco et al. 2017	Age: 22.7 ± 1.9 years Height: 1.76 ± 0.06 m Body mass: 75.8 ± 7.0 kg	VL20：N=12 VL40：N=10	Non-athletes (healthy trained individuals)	Physical education students; 1.5–4 years of resistance training; injury-free in the past 6 months; proficient in the full squat exercise.	20%VL vs 40%VL	8 weeks; 2 sessions/week.	Full squat training only; 70–85% 1RM; 3 sets/session; repetitions controlled by VL thresholds; 4-min inter-set rest.	CMJ 20m sprint

Galiano et al. 2020	VL5%: 22.1 ± 2.9 years, 175.1 ± 5.3 cm, 72.5 ± 11.3 kg; VL20%: 23.9 ± 3.0 years, 176.6 ± 3.5 cm, 75.7 ± 9.4 kg.	VL5：N=15 VL20：N=13	Non-athletes (healthy trained individuals)	⩾1.5 years of resistance training; no competitive sport background; proficient in the full squat exercise.	5%VL vs 20%VL	7 weeks; 2 sessions/week.	Full squat training only; fixed load at 50% 1RM; 3 sets/session; repetitions controlled by VL thresholds; 3-min inter-set rest.	CMJ 20m sprint
Myrholt et al. 2023	16 highly resistance-trained individuals (7 women and 9 men); LVL: 26.6 ± 3.5 years, 177.5 ± 6.5 cm, 76.3 ± 8.4 kg; HVL: 27.8 ± 3.6 years, 171.3 ± 8.6 cm, 74.3 ± 12.5 kg.	LVL：N=8 HVL：N=8	Highly resistance-trained individuals (advanced strength-trained population)	⩾12 months of systematic squat and bench press training; high strength level.	Squat: 20% VL vs. 40% VL; Bench press: 30% VL vs. 60% VL.	6 weeks; 3 sessions/week.	Free-weight squat + bench press training; 75 – 90% 1RM; 3 – 4-min inter-set rest; volume matched between groups; repetitions controlled by VL thresholds.	SJ
Rissanen et al. 2022	45 healthy young trained individuals (23 men and 22 women); Male VL20%: 183.6 ± 7.9 cm, 81.5 ± 8.0 kg; Male VL40%: 178.7 ± 5.9 cm, 82.3 ± 14.3 kg; Female VL20%: 167.0 ± 6.8 cm, 61.2 ± 4.6 kg; Female VL40%: 165.1 ± 7.1 cm, 60.1 ± 7.5 kg.	（Men） VL20：N=12 VL40：N=11 （Women） VL20：N=11 VL40：N=11	Non-athletes (recreational resistance-trained individuals)	⩾1 year of systematic back squat and bench press resistance training.	Squat + bench press: 20% VL vs. 40% VL Sex-specific comparisons.	8 weeks; 2 sessions/week (15 training sessions in total).	Smith-machine back squat + bench press training; 65 – 75% 1RM; 3-min rest; repetitions controlled by VL thresholds; maximal concentric effort; additional core training.	CMJ
Pérez-Castilla et al. 2017	VL10%: 22.2 ± 2.5 years, 73.9 ± 17.1 kg, 174.6 ± 5.9 cm; VL20%: 21.9 ± 1.7 years, 80.1 ± 15.2 kg, 176.7 ± 6.0 cm.	VL10：N=10 VL20：N=10	Non-athletes (healthy trained individuals)	Physical education students; ⩾2 years of recreational resistance training.	10%VL vs 20%VL	4 weeks; 2 sessions/week.	Loaded CMJ training; target mean propulsive velocity 1.20 m/s (~40% 1RM); 36 total repetitions; 4-min inter-set rest; repetitions controlled by VL thresholds	CMJ 15m sprint

Note: VL, velocity loss threshold; VBT, velocity-based resistance training; CMJ, countermovement jump; SJ, squat jump; RSI, reactive strength index; 1RM, one-repetition maximum; N, sample size; LVL, lower velocity loss threshold group; HVL, higher velocity loss threshold group. Age, height, and body mass are presented as mean ± standard deviation. Comparisons such as "10% VL vs. 20% VL" indicate comparisons between training groups with different velocity loss thresholds. Main outcomes included jump performance and linear sprint performance.

Electronic Supplementary Material Appendix S5
(Details of numerical data extracted from graphical presentation)

Table S5. Details of numerical data extracted from graphical presentation

Study	Outcome	Source	Extracted information	Verification	Effect-size revision
Rissanen et al. (2022)	Sex-specific CMJ performance	Figure 3A	Sex-specific relative changes in CMJ performance and corresponding dispersion values for men and women in the 20% and 40% VL groups	Independently checked by a second researcher against the original figure	Not required; extracted values were consistent with the original dataset

Note: CMJ, countermovement jump; VL, velocity loss. All other numerical data were obtained directly from the text or tables of the original studies.

Electronic Supplementary Material Appendix S6 (Risk of Bias in Included Studies)

Table S6. Risk of bias assessment of included studies

Study	D1 (Randomization process)	D2 (Deviations)	D3 (Missing outcome data)	D4 (Measurement)	D5 (Reporting)	Overall
Wang et al.（2026）	Some concerns	Some concerns	Low risk	Low risk	Some concerns	Some concerns
Rojas-Jaramillo et al.（2024）	Some concerns	Some concerns	Low risk	Low risk	Some concerns	Some concerns
Pareja-Blanco et al.（2016）	Some concerns	Some concerns	Some concerns	Low risk	Some concerns	Some concerns
Grazioli et al.（2023）	Some concerns	Some concerns	Low risk	Low risk	Some concerns	Some concerns
Grazioli et al.（2020）	Some concerns	Some concerns	Some concerns	Low risk	Some concerns	Some concerns
Rodríguez-Rosell et al.（2020）	Some concerns	Some concerns	Some concerns	Low risk	Some concerns	Some concerns
Rodríguez-Rosell et al.（2021）	Some concerns	Some concerns	Some concerns	Low risk	Some concerns	Some concerns
Pareja-Blanco et al.（2017）	Some concerns	Some concerns	Some concerns	Low risk	Some concerns	Some concerns
Galiano et al.（2020）	Some concerns	Some concerns	Some concerns	Low risk	Some concerns	Some concerns
Myrholt et al.（2023）	Some concerns	Some concerns	Low risk	Low risk	Some concerns	Some concerns
Rissanen et al.（2022）	Some concerns	Some concerns	Some concerns	Low risk	Some concerns	Some concerns
Pérez-Castilla et al.（2017）	Some concerns	Some concerns	Low risk	Low risk	Some concerns	Some concerns

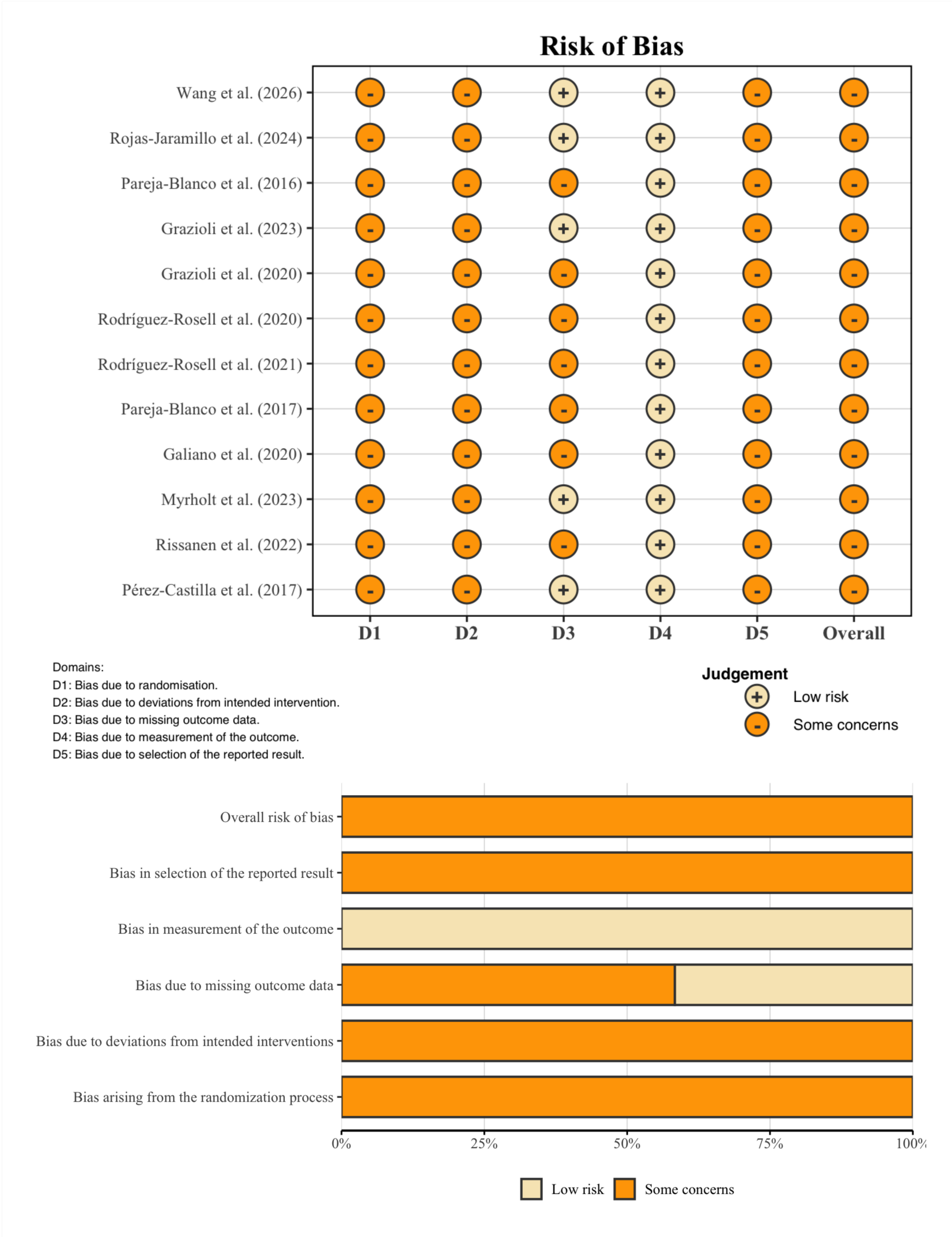

Fig. S1. Risk of bias assessment of the included studies.

Electronic Supplementary Material Appendix S7 (GRADE evidence profile)

Table S7. GRADE certainty assessment and reasons for downgrading for each outcome

Outcome	Studies	Risk of bias	Inconsistency	Indirectness	Imprecision	Publication bias	Quality	Reasons for downgrading / Explanation
CMJ	11	not serious	not serious	not serious	serious	undetected	Moderate	Downgraded one level for imprecision due to limited total sample size despite a statistically significant pooled effect.
SJ	3	not serious	not serious	not serious	very serious	undetected	Low	Downgraded two levels for very serious imprecision because only three studies were available, the total sample size was limited, and the confidence interval was wide and crossed the null effect.
30 m sprint	2	not serious	not serious	not serious	very serious	undetected	Low	Downgraded two levels for very serious imprecision because only two studies with a limited total sample size were available, resulting in substantial uncertainty despite the statistically significant pooled estimate.
20 m sprint	6	not serious	not serious	not serious	serious	undetected	Moderate	Downgraded one level for serious imprecision because the total information size was limited and the confidence interval crossed the null effect, leaving uncertainty regarding the presence and magnitude of the effect.
10 m sprint	5	not serious	not serious	not serious	serious	undetected	Moderate	Downgraded one level for imprecision due to limited total sample size.
jump performance	12	not serious	not serious	not serious	serious	undetected	Moderate	Downgraded one level for imprecision.
sprint performance	10	not serious	not serious	not serious	serious	undetected	Moderate	Downgraded one level for imprecision; possible small-study effects or publication bias should be considered based on asymmetry assessment.

Electronic Supplementary Material Appendix S8
(Sensitivity analyses using alternative assumed pre–post correlation coefficients)

Table S8. Sensitivity analyses using alternative assumed pre–post correlation coefficients

Assumed r	Studies	Effect sizes	Hedges' *g*	95% CI	*p*	95% PI	Level 2 I^2	Level 3 I^2	Total I^2
0.30	12	42	0.252	0.125–0.380	<0.001	0.125–0.380	<0.001%	0.001%	0.001%
0.50	12	42	0.293	0.165–0.421	<0.001	0.163–0.423	<0.001%	0.070%	0.070%
0.70	12	42	0.329	0.159–0.499	<0.001	−0.037–0.695	0.001%	12.708%	12.709%
0.90	12	42	0.461	0.190–0.732	<0.001	−0.352–1.274	0.003%	43.641%	43.643%

Note: r, assumed pre – post correlation coefficient; CI, confidence interval; PI, prediction interval. Positive effect sizes favor lower velocity loss thresholds. The primary analysis used r=0.50.

Electronic Supplementary Material Appendix S9 (Leave-one-out analysis)

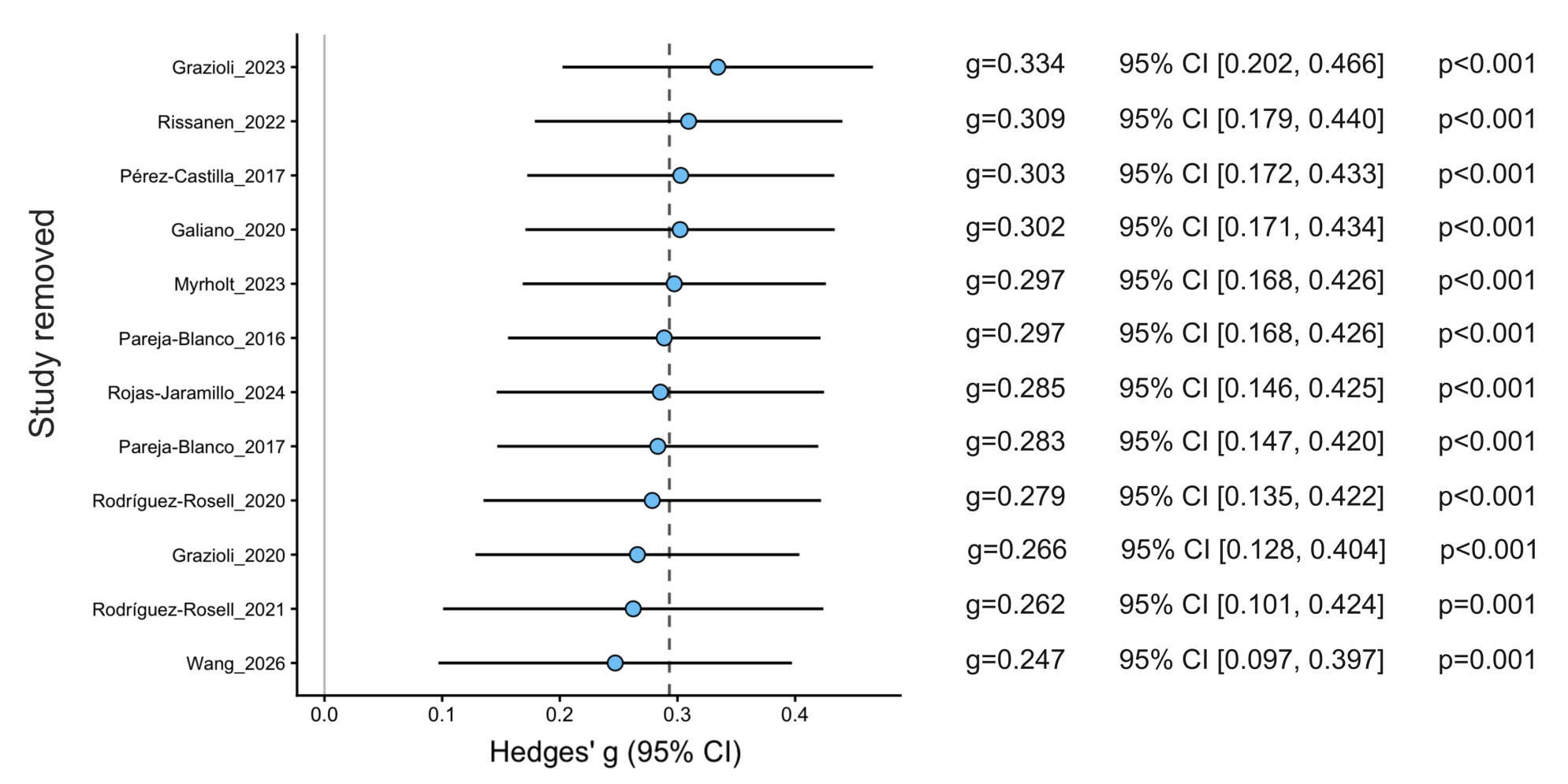

Figure 2. Leave-one-study-out sensitivity analysis of the pooled effect

Table S9. Leave-one-study-out sensitivity analysis of the pooled effect

Study removed	k	Studies	Hedges' g	95% CI	p value
Galiano_2020	40	11	0.302	[0.171, 0.434]	<0.001
Grazioli_2020	38	11	0.266	[0.128, 0.404]	<0.001
Grazioli_2023	39	11	0.334	[0.202, 0.466]	<0.001
Myrholt_2023	41	11	0.297	[0.168, 0.426]	<0.001
Pareja-Blanco_2016	40	11	0.289	[0.156, 0.422]	<0.001
Pareja-Blanco_2017	40	11	0.283	[0.147, 0.420]	<0.001
Pérez-Castilla_2017	40	11	0.303	[0.172, 0.433]	<0.001
Rissanen_2022	40	11	0.309	[0.179, 0.440]	<0.001
Rodríguez-Rosell_2020	39	11	0.279	[0.135, 0.422]	<0.001
Rodríguez-Rosell_2021	33	11	0.262	[0.101, 0.424]	0.001
Rojas-Jaramillo_2024	39	11	0.285	[0.146, 0.425]	<0.001
Wang_2026	33	11	0.247	[0.097, 0.397]	0.001

Electronic Supplementary Material Appendix S10
(Sensitivity analyses using alternative model specifications)

Table S10. Sensitivity analyses using alternative model specifications

Model specification	Studies	Effect sizes	Hedges' *g*	95% CI	*p*	95% PI	Total I^2
Three-level random-effects model	12	42	0.293	0.166–0.421	<0.001	0.166–0.421	<0.001%
Study-level random-effects model	12	42	0.293	0.166–0.421	<0.001	0.166–0.421	<0.001%
Conventional random-effects model	12	42	0.293	0.166–0.421	<0.001	0.166–0.421	0.000%

Note: The study-level random-effects model included a random intercept at the study level but did not separately estimate an effect-size-level random component. The conventional random-effects model treated the 42 effect sizes as statistically independent and was used only as a sensitivity analysis. The primary analysis was the three-level random-effects model.
